# Functional Activation of the Flagellar Type III Secretion Export Apparatus

**DOI:** 10.1371/journal.pgen.1005443

**Published:** 2015-08-05

**Authors:** Andrew M. Phillips, Rebecca A. Calvo, Daniel B. Kearns

**Affiliations:** Department of Biology, Indiana University, Bloomington, Indiana, United States of America; Universidad de Sevilla, SPAIN

## Abstract

Flagella are assembled sequentially from the inside-out with morphogenetic checkpoints that enforce the temporal order of subunit addition. Here we show that flagellar basal bodies fail to proceed to hook assembly at high frequency in the absence of the monotopic protein SwrB of *Bacillus subtilis*. Genetic suppressor analysis indicates that SwrB activates the flagellar type III secretion export apparatus by the membrane protein FliP. Furthermore, mutants defective in the flagellar C-ring phenocopy the absence of SwrB for reduced hook frequency and C-ring defects may be bypassed either by SwrB overexpression or by a gain-of-function allele in the polymerization domain of FliG. We conclude that SwrB enhances the probability that the flagellar basal body adopts a conformation proficient for secretion to ensure that rod and hook subunits are not secreted in the absence of a suitable platform on which to polymerize.

## Author Summary

Bacteria build needle-like injectsomes to secrete toxins into host cells and build propeller-like flagella to swim through their environment using a molecular machine called the type III secretion system (T3SS). Both the injectisome and the flagellum are large self-assembling complexes and regulation of the T3SS ensures that proteins are secreted sequentially for proper structure and function. Here we report genetic and cytological data that the SwrB protein of *Bacillus subtilis* helps the base of the flagellum adopt a completed conformation which in turn activates the enclosed T3SS to export proteins for the next stage of flagellar assembly. Thus SwrB presents a novel mechanism to supervise an early structural checkpoint regulating machine assembly. Targeting functional regulators like SwrB could inhibit T3SS-based strategies of pathogens.

## Introduction

Some bacteria swim through liquid and swarm over surfaces by synthesizing trans-envelope nanomachines called flagella. Flagella spontaneously self-assemble from over twenty separate proteins thought to be organized into three structural domains called the basal body, the hook and the filament [1–3]. The basal body is composed of a ring of a transmembrane protein called FliF that surrounds a membrane-embedded, dedicated type III secretion export apparatus [4–7]. Beneath the basal body sits the FliG rotor that interacts with the proton-conducting stators to generate torque, as well as the C-ring proteins FliM and FliN that control the direction of flagellar rotation [8,9]. The export apparatus secretes subunits of the drive-shaft rod that transits the peptidoglycan, the universal joint hook, and the long helical filament. When the basal body rotates, rotational energy is transmitted through the rod and the hook to turn the filament that generates propulsion like a propeller. Central to flagellar assembly is the control of the flagellar type III secretion export apparatus.

Type III secretion export apparati are housed within both the flagellar basal body and the evolutionarily-related “injectisome” used by various pathogenic bacteria to secrete toxins directly into eukaryotic host cells [10,11]. Recent cytological evidence suggests that the export apparatus is likely the first substructure to form and serves as the nucleation point for subsequent flagellar/injectisome basal body assembly [7,12,13]. The export apparatus requires a conserved set of 5 transmembrane proteins: FliP, FliQ, FliR, FlhA, and FlhB [6,14]. FlhA is important for the recognition of secretion substrates and FlhB controls the substrate specificity of the export apparatus [15–18]. The roles of FliP, FliQ, and FliR are poorly understood, but presumably these proteins either transduce the proton motive force that powers protein export or serve as the export channel [19,20]. Flagellar export systems differ from injectisome export systems in that flagella require a sixth transmembrane protein FliO that appears to function as a regulator [21,22].

The flagellar export apparatus secretes two classes of proteins considered “early” and “late” depending on the temporal order in which they are secreted. Early class flagellar structural components like the rod and the hook subunits are secreted first, and are recognized by information encoded within the N-terminus of the secreted protein [23–25]. Late class subunits, which include the hook-associated proteins and flagellin, are ushered by chaperones and are secreted only after a substrate specificity switch occurs within the export apparatus in response to hook completion [17,26–28]. Furthermore, the expression of the genes encoding the late class secretion substrates is inhibited by the anti-sigma factor FlgM prior to hook completion. Once the hook is complete and the substrate specificity switch occurs, FlgM is exported, its cognate sigma factor is liberated, and late class flagellar genes are expressed [29,30]. Thus, the activity of the export apparatus is morphogenetically coupled to flagellar structure so as to govern subsequent flagellar gene expression and assembly. Finally, there is evidence for another morphogenetic coupling event that precedes hook completion as mutants defective in the cytoplasmic flagellar rotor FliG and C-ring components FliM and FliN have flagellar type III secretion defects [31–37].

Here we provide further evidence for an early morphogenetic checkpoint in flagellar assembly whereby completion of the flagellar basal body and the single pass transmembrane protein SwrB of *Bacillus subtilis* functionally activate the flagellar type III export apparatus to become secretion proficient. Mutants defective for SwrB assemble a reduced number of flagella due to a reduced frequency of hook assembly, and a secretion defect was implicated as many genetic suppressors increased the abundance of the secretion apparatus component FliP. We show that the proficiency of the export apparatus for hook subunit secretion was coupled to the conformation of the basal body as C-ring mutants phenocopied the absence of SwrB. Furthermore, overexpression of SwrB rescued hook assembly to cells defective for FliG, and FliG gain-of-function alleles suppressed the absence of SwrB. We conclude that SwrB chaperones the formation of a completed basal body conformation thereby activating the flagellar type III export apparatus for the secretion of the hook, and likely rod, structural subunits.

## Results

### SwrB is required for hook, but not basal body (C-ring) assembly

**Fig 1:**
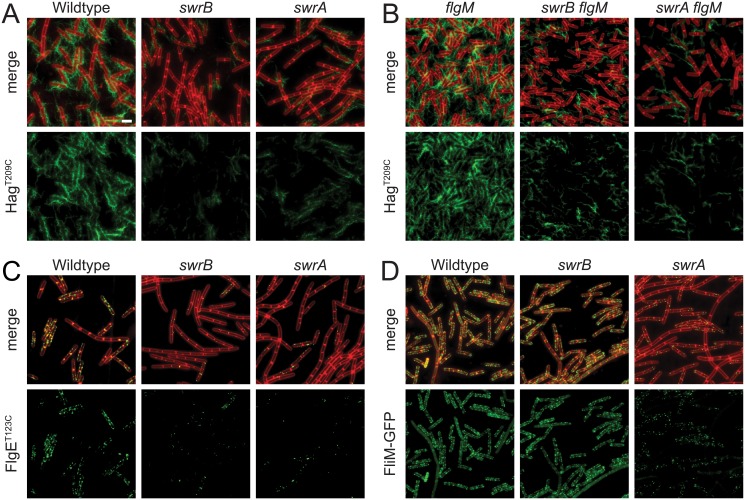
SwrB is required for assembly of hooks but not basal bodies. Panels A and B) Fluorescence micrographs of the flagellar filament (Hag^T209C^) in the indicated genetic backgrounds. Membranes were stained with FM4-64 and false colored red. Flagella were stained with maleimide alexa fluor 488 and false colored green. The following strains were used to generate these panels: wild type (DS1916), *swrA* (DS9515), *swrB* (DS9319), *flgM* (DK486), *swrA flgM* (DK487), *swrB flgM* (DS488). Panel C) Fluorescence micrographs of the flagellar hook (FlgE^T123C^) in the indicated genetic backgrounds. Membranes were stained with FM4-64 and false colored red. Hooks were stained with maleimide alexa fluor 488 and false colored green. The following strains were used to generate this panel: wild type (DS7673), *swrA* (DK480), *swrB* (DK478). Panel D) Fluorescence micrographs of flagellar basal bodies (FliM-GFP) in the indicated genetic backgrounds. Membranes were stained with the membrane stain FM4-64 and false colored red. FliM-GFP puncta were false colored green. The following strains were used to generate this panel: wild type (DS8521), *swrA* (DS8600), *swrB* (DK479). Scale bar is 4 μm.

SwrB is a single-pass transmembrane protein of unknown function that is required for swarming motility in *B*. *subtilis* [38,39]. Swarming motility over a solid surface requires an increase in the number of flagella relative to swimming in liquid, and mutants defective in the master regulator of flagellar biosynthesis, SwrA, have reduced flagellar number and are unable to swarm [40–42]. To determine whether cells mutated for SwrB have a defect in flagellar filament number, a variant of the flagellar filament protein Hag that could be labeled with a fluorescent dye (Hag^T209C^) was introduced to the wild type, *swrB*, and *swrA* mutant backgrounds [43]. Cells mutated for SwrB appeared to have fewer filaments than wild type and resembled cells mutated for SwrA (Fig 1A). We conclude that SwrB is required for the assembly of wild type numbers of flagellar filaments and we infer that the reduction in filament number accounts for the swarming defect of a *swrB* mutant.

**Fig 2:**
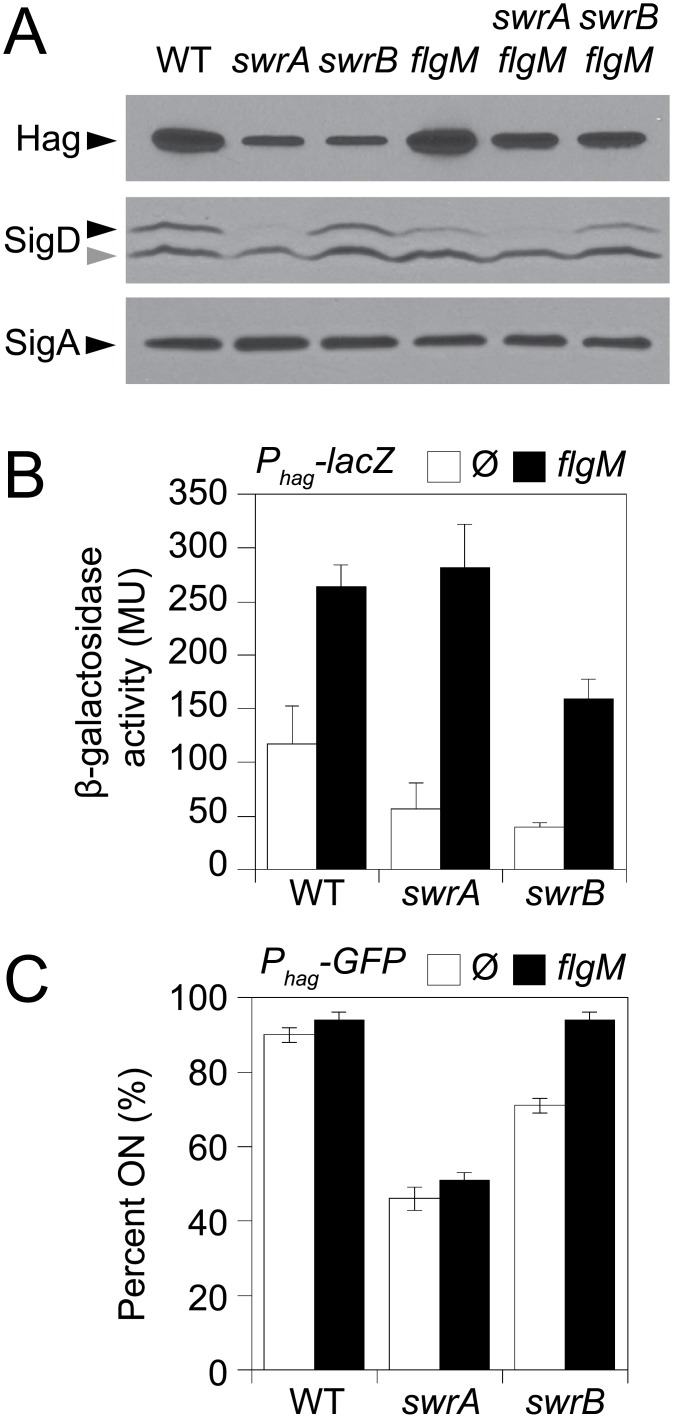
SwrB increases σ^D^ activity but not σ^D^ protein levels. A) Western blot analysis of whole cell lysates from the indicated genetic backgrounds probed with anti-Hag, anti-SigD, and anti-SigA primary antibodies. Black carets indicate antibody specific targets. Gray caret indicates a non-specific cross-reacting band recognized by the anti-SigD antibody. Strains used to generate this panel: wild type (DS908), *swrA* (DS4015), *swrB*, (DS4040), *flgM* (DS4264), *swrA flgM* (DS4034), *swrB flgM* (DS4090). B) β-galactosidase assays of *P*_*hag*_ *-lacZ* transcriptional activity expressed in Miller Units in the indicated genetic backgrounds. The Ø symbol indicates that no further genetic modification was included whereas “*flgM*” indicates the introduction of a *flgM* mutation to the indicated genetic background. β-galactosidase values presented in S3 Table. The following strains were used to generate this panel: wild type (DS9461), *flgM* (DK313), *swrA* (DK288), *swrA flgM* (DK318), *swrB* (DK289), *swrB flgM* (DK319). C) The frequency of fluorescent “ON” cells expressing *P*_*hag*_ *-GFP* in the indicated genetic backgrounds. Over 600 cells were counted per strain in triplicate to obtain average percentages and standard deviations. The Ø symbol indicates that no further genetic modification was included whereas “*flgM*” indicates the introduction of a *flgM* mutation to the indicated genetic background. Sample raw data images used to generate the percentages are presented in S1 Fig. Strains used to generate this panel: wild type (DS908), *swrA* (DS4015), *swrB* (DS4040), *flgM* (DS4264), *swrA flgM* (DS4034), and *swrB flgM* (DS4090).

Consistent with a reduction in flagellar filament assembly, cells lacking either SwrB or SwrA showed a reduced level of the flagellar filament protein, Hag, by Western blot analysis (Fig 2A). The reduction in Hag protein was likely due to reduced transcription of the *hag* gene as cells mutated for either SwrB or SwrA showed lower expression from a reporter in which the *hag* promoter (*P*_*hag*_) was fused to the *lacZ* gene encoding β-galactosidase (*P*_*hag*_ *-lacZ*) (Fig 2B, white bars), and a reduced frequency of cells expressing a reporter in which the *P*_*hag*_ promoter was fused to green fluorescent protein (GFP) (*P*_*hag*_ *-gfp*) (Fig 2C, white bars, and S1 Fig) [39,41]. The *P*_*hag*_ promoter is transcribed by RNA polymerase and the alternative sigma factor, σ^D^ [44]. Whereas cells mutated for SwrA exhibited a reduced level of σ^D^ protein, cells mutated for SwrB exhibited a level of σ^D^ protein comparable to the wild type (Fig 2A). We conclude that the absence of SwrB resulted in reduced expression of the *hag* gene but unlike the absence of SwrA, the defect occurred downstream of σ^D^ protein levels.

One reason that *hag* expression might be reduced in the SwrB mutant despite wild type levels of σ^D^ is due to enhanced inhibition of σ^D^ by its cognate anti-sigma factor FlgM [45–48]. Consistent with enhanced inhibition by FlgM, mutation of FlgM increased the magnitude of expression of the *P*_*hag*_ *-lacZ* reporter (Fig 2B, black bars), and increased the frequency of expression of the *P*_*hag*_ *-GFP* reporter in the wild type and *swrB* mutant backgrounds (Fig 2C, black bars, and S1 Fig). The SwrA protein controls the frequency of cells that have σ^D^ protein levels above a critical threshold upstream of FlgM regulation [49,50] and thus simultaneous mutation of both FlgM and SwrA increased expression magnitude in a subpopulation (Fig 2B) but did not increase the frequency of σ^D^ activity (Fig 2C). Finally, mutation of FlgM, did not appear to increase flagellar filament number in the absence of either SwrB or SwrA (Fig 1B) despite a modest increase in flagellin protein levels (Fig 2A). We conclude that enhanced inhibition of σ^D^ by FlgM was a consequence and not a cause of the reduction of flagellar number in the *swrB* mutant.

**Fig 3:**
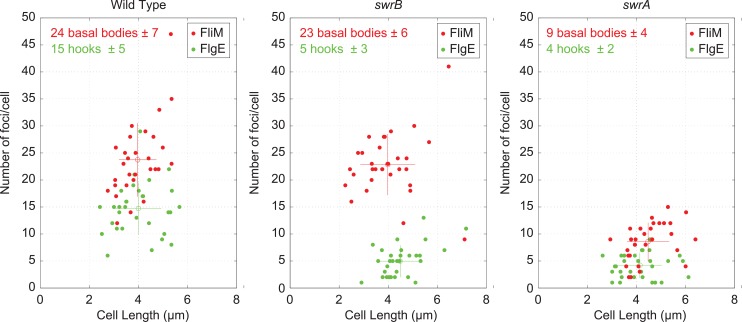
Cytological quantification of flagellar hooks and basal bodies. 3-D SIM microscopy and Imaris software was used count puncta per cell for either basal bodies (red dots, FliM-GFP) or hooks (green dots/maleimide stained FlgE^T123C^) relative to cell length on 30 individual cells each of the wild type (DS8521/DS7673), *swrB* (DK479/DK478) and *swrA* (DS8600/DK480) backgrounds. Open circles represent the puncta number and length averages of the matching color, and vertical and horizontal bars indicate the standard deviations respectively.

FlgM inhibition of σ^D^ activity is enhanced when cells are defective in flagellar hook synthesis, and a defect in flagellar hook synthesis would also account for the reduction in flagellar filaments observed in the SwrB mutant [51,52]. To measure flagellar hook numbers, a variant of the flagellar hook protein FlgE that could be labeled with a fluorescent dye (FlgE^T123C^) was introduced to the wild type, *swrB*, and *swrA* mutant backgrounds [51]. Cells mutated for *swrB* appeared to have fewer hooks than the wild type and instead resembled the reduced numbers of hooks in cells mutated for *swrA* (Fig 1C). To count flagellar hooks, 3D structured illumination microscopy (3D-SIM) was conducted on hook-stained cells of each genetic background and each individual cell was expressed as a point on a scatter plot representing the number of hooks versus cell length (Fig 3, green symbols). Whereas wild type cells had an average of 15 hooks, *swrB* and *swrA* mutants had an average of 5 and 4 hooks per cell, respectively. We conclude that the *swrB* mutant was defective in hook assembly and as a result exhibited enhanced inhibition of σ^D^ by FlgM.

Because hook assembly depends on flagellar basal body synthesis, the reduction in hook numbers observed in a *swrB* mutant could be due to a commensurate reduction in basal body number. To measure flagellar basal body synthesis, a translational fusion of GFP to the C-ring protein FliM (*amyE*::*P*_*fla/che*_ *-fliM-GFP*) was introduced to the wild type, *swrB*, and *swrA* mutant backgrounds [53]. Cells mutated for *swrB* appeared to have wild type numbers of flagellar C-rings, whereas cells mutated for *swrA* appeared to have a reduction in flagellar basal body number, as previously reported (Fig 1D) [53]. To count flagellar C-rings 3D-SIM was conducted on cells of each genetic background and each individual cell was expressed as a point on a scatter plot representing the number of basal bodies versus cell length (Fig 3, red symbols). On average, wild type had 24 C-rings, the *swrB* mutant had 23 C-rings, and the *swrA* mutant had 9 C-rings per cell. We conclude that SwrB and SwrA increased flagellar numbers at two different steps in flagellar assembly by promoting hook and C-ring (basal body) assembly respectively. We further conclude that SwrB somehow promoted hook assembly at a step downstream of the incorporation of FliM into the basal body.

### SwrB activates the flagellar export apparatus

**Fig 4:**
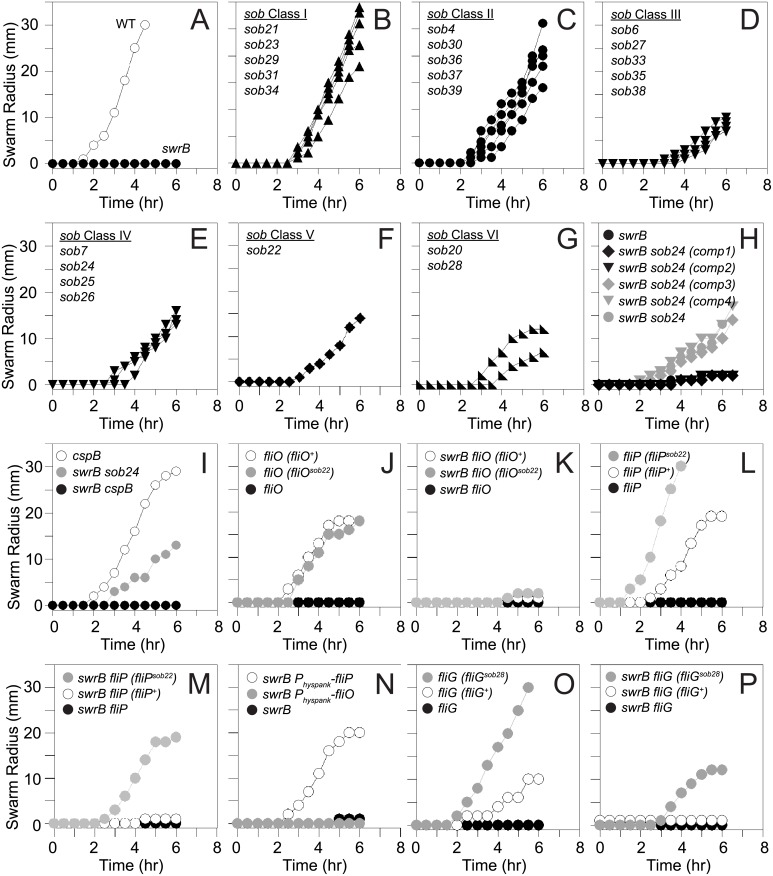
Suppressors of *swrB* (*sob*) mutants restore swarming motility in the absence of SwrB. Quantitative swarm expansion assays of the indicated genetic backgrounds. Genotypes in parentheses indicate complementation constructs. The following strains were used the generate panels: A) wild type (3610), *swrB* (DS234), B) *swrB sob21* (DS9148), *swrB sob23* (DS9150), *swrB sob29* (DS9156), *swrB sob31* (DS9158), *swrB sob34* (DS9817), C) *swrB sob4* (DS4633), *swrB sob30* (DS9157), *swrB sob36* (DS9819), *swrB sob37* (DS9831), *swrB sob39* (DS9846), D) *swrB sob6* (DS7063), *swrB sob27* (DS9154), *swrB sob33* (DS9816), *swrB sob35* (DS9818), *swrB sob38* (DS9845), E) *swrB sob7* (DS7064) *swrB sob24* (DS9151), *swrB sob25* (DS9152), *swrB sob26* (DS9153), F) *swrB sob22* (DS9149), G) swrB *sob20* (DS9147), *swrB sob28* (DS9155), H) *swrB* (DS234), *swrB sob24* (comp1) (DK2015), *swrB sob24* (comp2) (DK2017), *swrB sob24* (comp3) (DK2016), *swrB sob24* (comp4) (DK2018), *swrB sob24* (DS9151) I) *cspB* (DK1905), *swrB sob24* (DS9151), *swrB cspB* (DK1937), J) *fliO* (DS6468), *fliO (fliO* ^*+*^ *)* (DK52), *fliO (fliO* ^*sob22*^ *)* (DK53), K) *swrB fliO* (DK54), *swrB fliO (fliO* ^*+*^ *)* (DK65), *swrB fliO (fliO* ^*sob22*^ *)* (DK66), L) *fliP* (DS7119), *fliP (fliP* ^*+*^ *)* (DK577), *fliP (fliP* ^*sob22*^ *)* (DK579), M) *swrB fliP* (DK519), *swrB fliP (fliP* ^*+*^ *)* (DK572), *swrB fliP (fliP* ^*sob22*^ *)* (DK574), N) *swrB* (DS234), *swrB amyE*::*P*_*hyspank*_ *-fliO* (DK1230), *swrB amyE*::*P*_*hyspank*_ *-fliP* (DK601) grown in the presence of 1 mM IPTG, O) *fliG* (DS7357), *fliG* (*fliG* ^*+*^) (DK737), *fliG* (*fliG* ^*sob28*^) (DK568), P) *swrB fliG* (DK584), *swrB fliG* (*fliG* ^*+*^) (DK738), *swrB fliG* (*fliG* ^*sob28*^) (DK598). Each data point is the average of three replicates.

**Fig 5:**
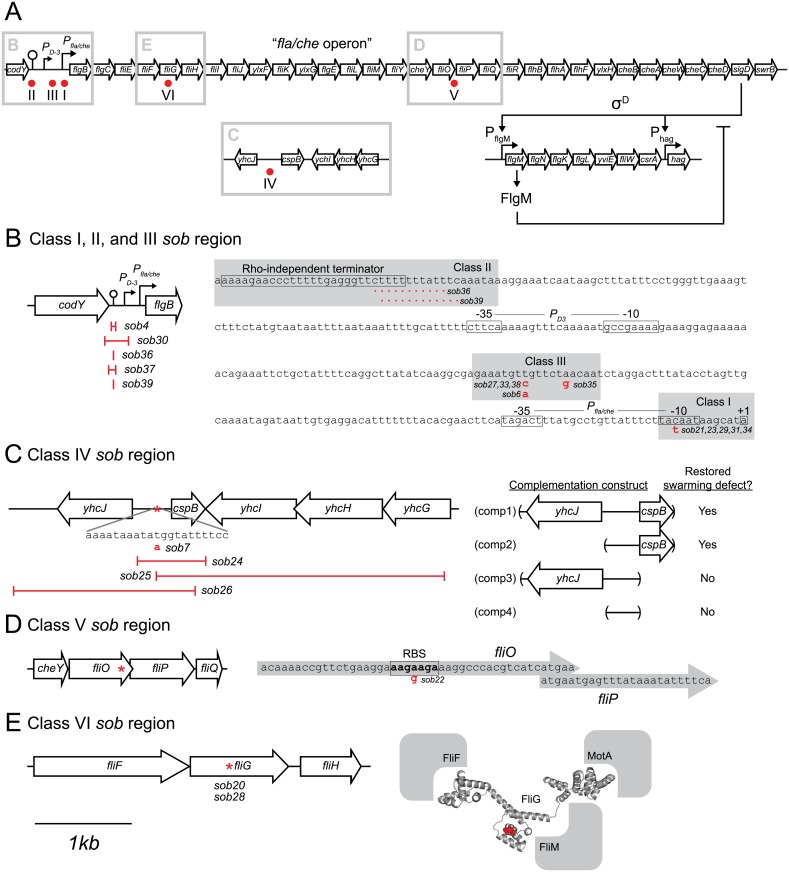
Genetic maps indicating the location of the *sob* suppressors. A) Map of the location of the *sob* mutant classes as indicated by red dots. Gray boxes indicate the enlarged regions emphasized in panels B-E. For all panels, open arrows indicate open reading frames, bent arrows indicate promoters, lollipops indicate Rho-independent terminators, and “I” bars indicate the boundaries of genetic deletions. The map in panel A is not to scale. The maps in panels B-E share the 1 kb scale bar as indicated beneath panel E. B) Class I, II, III *sob* mutant locations. Left, cartoon of the *P*_*fla/che*_ promoter region indicating the location of the class II *sob* deletion boundaries. Right, sequence of the *P*_*fla/che*_ promoter region with *sob* Class I and III point mutant locations indicated with gray boxes and annotated in red letters. *sob* class II deletions annotated as red dotted lines. Important genetic landmarks including the *codY* terminator sequence, the *P*_*D3*_ promoter elements, the *P*_*fla/che*_ promoter elements, and the *fla/che* operon transcriptional start site are boxed and labeled. C) Class IV *sob* mutant locations. Left, cartoon of the *cspB* upstream intergenic region. Boundaries of deletions and the location of a single base pair mutation in red. Right, cartoon of the boundaries of the complementation constructs generated for the *swrB sob24* allele. Results summarized and interpreted based on swarm assay presented in Fig 4H. D) Class V *sob* mutant location. Left, cartoon of the *fliO*/*fliP* genetic region and the location of the *sob* mutation indicated by a red asterisk. Right, sequence of the 3’ end of the *fliO* gene and the 5’ end of the *fliP* gene. Putative internal RBS sequence is boxed and labeled. The location of the *sob* class V mutation indicated by a red letter. E) Class VI *sob* mutant locations. Left, cartoon of the *fliG* genetic region and the location of the *sob* mutation indicated by a red asterisk. Right, structure of the *Aquifex aeolicus* FliG protein with the FliG^Q132^ residue which was mutated to an asparagine in the *sob* mutants [64]. The predicted locations of the FliF, MotA, and FliM proteins that interact with FliG are indicated in gray.

To determine how SwrB regulates hook assembly, spontaneous suppressor mutations were isolated that restored swarming motility to a *swrB* mutant. When a *swrB* mutant was inoculated in the center of a swarm agar plate, cells initially grew as a tight central colony and, unlike the wild type, failed to spread from the inoculum origin (Fig 4A). However, after 24 hours of incubation, flares of cells that had regained the ability to swarm emerged from the central colony and cells from these flares were clonally isolated. Twenty-four spontaneous *sob* (suppressor of *swr* *B*) mutants were independently isolated. Each suppressor was validated by PCR length polymorphism to confirm the presence of the *swrB* mutant allele. A combination of candidate gene sequencing, phage transduction linkage mapping, and Illumina whole-genome sequencing was used to identify the location of each of the *sob* suppressor mutations. Based on the swarm behavior and location of the suppressor mutations, the *sob* alleles were divided into six classes and analyzed separately (Table 1 and Figs 4B–4G and 5A).

**Table 1:** Suppressor of *swrB* (*sob*) alleles.

suppressor	strain	genotype
Class I—improved *fla/che* consensus		
sob21	DS9148	*P*_*fla/che*_ TACAAT > TA**T**AAT (-10 element)
sob23	DS9150	*P*_*fla/che*_ TACAAT > TA**T**AAT (-10 element)
sob29	DS9156	*P*_*fla/che*_ TACAAT > TA**T**AAT (-10 element)
sob31	DS9158	*P*_*fla/che*_ TACAAT > TA**T**AAT (-10 element)
sob34	DS9817	*P*_*fla/che*_ TACAAT > TA**T**AAT (-10 element)
Class II—*codY* terminator deletes		
sob4	DS4633	45 bp deletion spanning *codY* rho-independent terminator
sob30	DS9157	245 bp deletion spanning *codY* rho-independent terminator
sob36	DS9819	11 bp deletion spanning *codY* rho-independent terminator
sob37	DS9831	50 bp deletion spanning *codY* rho-independent terminator
sob39	DS9846	12 bp deletion spanning *codY* rho-independent terminator
Class III—mutations upstream of *P*_*fla/che*_		
sob6	DS7063	T > A upstream of the *P*_*fla/che*_ promoter at position -110
sob27	DS9154	T > C upstream of the *P*_*fla/che*_ promoter at position -110
sob33	DS9816	T > C upstream of the *P*_*fla/che*_ promoter at position -110
sob35	DS9818	A > G upstream of the *P*_*fla/che*_ promoter at position -104
sob38	DS9845	T > C upstream of the *P*_*fla/che*_ promoter at position -110
Class IV—Mutations of intergenic region upstream of *cspB*		
sob7	DS7064	G > A at 5’ end of predicted ORF upstream of *cspB*
sob24	DS9151	706 bp deletion spanning *cspB*
sob25	DS9152	2975 bp deletion spanning *cspB*
sob26	DS9153	1875 bp deletion spanning *cspB*
Class V—*fliP* enhanced translation		
sob22	DS9149	AGAAGA > AG**G**AGA improved *fliP* RBS (FliO^K212E^)
Class VI—FliG assembly domain		
sob20	DS9147	CAG > C**G**G (FliG^Q132R^)
sob28	DS9155	CAG > C**G**G (FliG^Q132R^)

#### Class I–Improved *P*_*fla/che*_ consensus

The *sob* class I alleles were identified by PCR amplification and directed sequencing of the *P*_*fla/che*_ promoter region, as the *P*_*fla/che*_ promoter has been found to be a common site for suppressors of motility defects [41,54]. Five independently-isolated *sob* alleles contained the same single base pair point mutation that changed the TACAAT -10 element of *P*_*fla/che*_ promoter to a consensus TATAAT sequence (Table 1 and Fig 5B). We note that the same kinds of mutations increased *P*_*fla/che*_ promoter activity in previously-reported suppressors of *swrA* (*soa*) [41], in 8 out of 23 newly-isolated *soa* alleles (S2 Fig and Table 2), and in suppressors of a dominant *degU32(Hy)* allele (*dhs*) [54]. We infer that one way to suppress a defect in SwrB is by increasing *P*_*fla/che*_ promoter activity.

**Table 2:** Suppressor of *swrA* (*soa*) alleles.

suppressor	strain	genotype
Class I—improved P_*fla/che*_ promoter		
soa3 (soa^C^)	DS725	TACAAT > TA**T**AAT (-10 element) [41]
soa4 (soa^D^)	DS726	TACAAT > TA**T**AAT (-10 element) [41]
soa5	DK1056	T > G upstream of the *P*_*fla/che*_ -10 element at position -16 to *fla/che* transcript start
soa9	DK1060	TACAAT > TA**T**AAT (-10 element)
soa11	DK1062	TACAAT > TA**T**AAT (-10 element)
soa13	DK1064	TACAAT > TA**T**AAT (-10 element)
soa17	DK1068	TACAAT > TA**T**AAT (-10 element)
soa19	DK1070	TACAAT > TA**T**AAT (-10 element)
soa23	DK1074	C > G upstream of the *P*_*fla/che*_ -10 element at position -14 to *fla/che* transcript start
soa25	DK1076	TAGACT > T**T**GACT (-35 element)
Class II—*codY* terminator mutations		
soa6	DK1057	227 bp deletion spanning *codY* Rho-independent terminator
soa8	DK1059	G > C in *codY* terminator at position -291 to *fla/che* transcript start
soa12	DK1063	13 bp deletion spanning *codY* Rho-independent terminator
soa14	DK1065	A > T in *codY* terminator at position -294 to *fla/che* transcript start
soa16	DK1067	A > T in *codY* terminator at position -294 to *fla/che* transcript start
soa18	DK1069	1920 bp deletion spanning *codY* Rho-independent terminator
soa21	DK1072	12 bp deletion spanning *codY* Rho-independent terminator
soa22	DK1073	245 bp deletion spanning *codY* Rho-independent terminator
soa24	DK1075	196 bp deletion spanning *codY* Rho-independent terminator
soa26	DK1077	66 bp deletion spanning *codY* Rho-independent terminator
soa28	DK1079	227 bp deletion spanning *codY* Rho-independent terminator
soa20	DK1071	420 bp deletion spanning *codY* Rho-independent terminator
soa27	DK1078	227 bp deletion spanning *codY* Rho-independent terminator
Class III—point mutations upstream of *P*_*fla/che*_		
soa7	DK1058	A > G upstream of the *P*_*fla/che*_ promoter at position -57 to *fla/che* transcript start
soa15	DK1066	G > A upstream of the *P*_*fla/che*_ promoter at position -225 to *fla/che* transcript start

**Fig 6:**
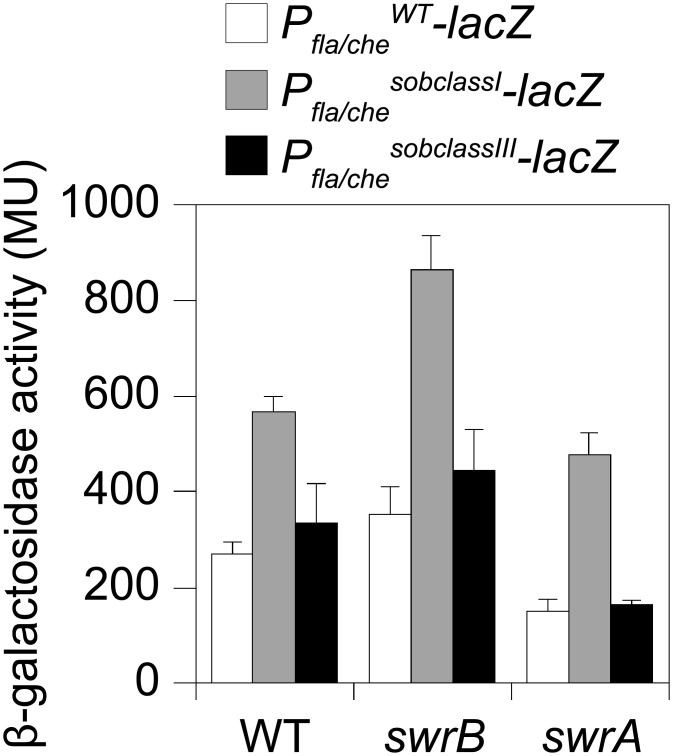
Class I but not Class III *sob* alleles in the *P*_*fla/che*_ promoter region increase *P*_*fla/che*_ expression. β-galactosidase assays of *lacZ* gene expression under the control of WT (open bars), Class I *sob21* (gray bars), or Class III *sob6* (black bars) *P*_*fla/che*_ promoter regions. Each reporter was expressed in either WT, *swrB*, or *swrA* mutant backgrounds as indicated. The following strains were used to generate this panel: *amyE*::*P*_*flache*_^*WT*^ *-lacZ* (DS793), *amyE*::*P*_*flache*_^*sob21*^ *-lacZ* (DS1426), *amyE*::*P*_*flache*_^*sob6*^ *-lacZ* (DS9120), *swrB amyE*::*P*_*flache*_^*WT*^ *-lacZ* (DK285), *swrB amyE*::*P*_*flache*_^*sob21*^ *-lacZ* (DK1130), *swrB amyE*::*P*_*flache*_^*sob6*^ *-lacZ* (DK1117), *swrA amyE*::*P*_*flache*_^*WT*^ *-lacZ* (DK284), *swrA amyE*::*P*_*flache*_^*sob21*^ *-lacZ* (DK1129), *swrA amyE*::*P*_*flache*_^*sob6*^ *-lacZ* (DK1116). Error bars are the standard deviations of three replicates. β-galactosidase values presented in S4 Table.

To directly test the consequence of a *sob* class I allele in a *swrB* mutant background, the *P*_*fla/che*_ promoter region was cloned either from wild type or a class I *sob21* allele upstream of a promoterless *lacZ* gene and inserted at an ectopic locus (*amyE*::*P*_*fla/che*_^*WT*^ *-lacZ* and *amyE*::*P*_*fla/che*_^*sobclassI*^ *-lacZ*). Consistent with previous reports, the improved -10 promoter element of the *sob* class I allele increased expression of *P*_*fla/che*_ in wild type, *swrB*, and *swrA* mutant backgrounds relative to the wild type promoter (Fig 6, compare white and gray bars within strains). Unlike mutation of SwrA, however, mutation of SwrB did not reduce the expression of the *P*_*fla/che*_ wild type reporter in an otherwise wild type genetic background (Fig 6, compare white bars between strains). We conclude that although SwrB does not normally act to increase the expression of the P_*fla/che*_ promoter, increased transcriptional activity from the P_*fla/che*_ promoter nonetheless compensates for the absence of SwrB.

**Fig 7:**
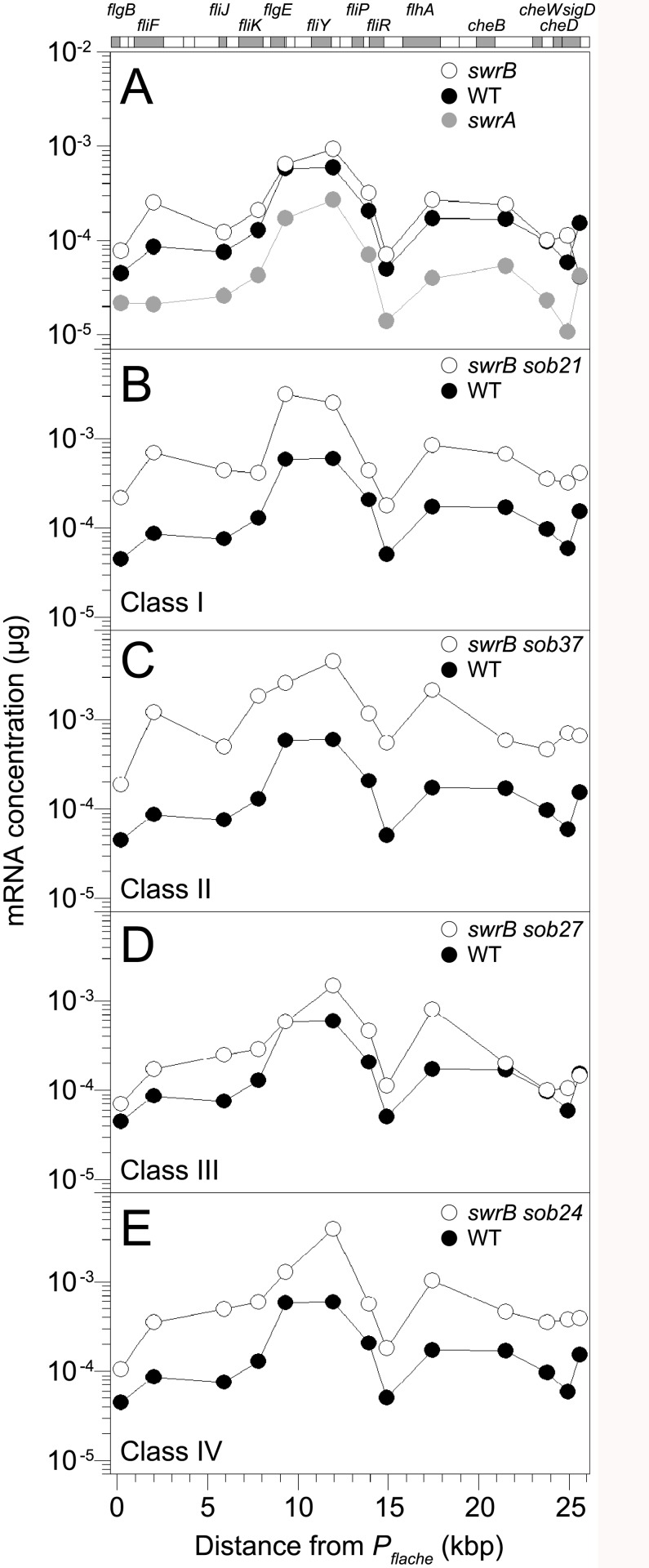
Some *sob* classes increase *fla/che* operon expression. QRT-PCR analysis of *fla/che* operon transcript levels at various positions in the operon. At the top of the graphs is a schematic of the *fla/che* operon where individual genes are drawn to scale and represented as boxes. Shaded boxes correspond to the locations of data points immediately below. Data points express the mRNA concentration corresponding to the gene probed by QRT-PCR at the kb distance *fla/che* operon transcriptional start site. Each data point is the average of three independently harvested RNA samples from whole cell lysates normalized to the transcript abundance of the constitutively expressed *sigA* gene. For each graph, the same wild type dataset from strain 3610 (closed circles) was used as a comparator. The following strains were used to generate this panel: A) *swrA* (DS2415), *swrB* (DS234), B) *swrB sob21* (Class I, DS9148), C) *swrB sob37* (Class II, DS9831), D) *swrB sob27* (Class III, DS9154), and E) *swrB sob24* (Class IV, DS9151).

Expression from the *P*_*fla/che*_ promoter is thought to control transcript abundance of the enitre *fla/che* operon [49,50,55,56]. To determine *fla/che* transcript levels in the absence of SwrB, QRT-PCR was conducted at various positions along the length of operon. Consistent with results observed with the *P*_*fla/che*_ *-lacZ* reporter assay, mutation of SwrA resulted in a reduction in *fla/che* operon transcript abundance but mutation of SwrB did not (Fig 7A). When the *sob21*-improved *P*_*fla/che*_ -10 element class I allele was present at the native site in the chromosome, transcript levels increased from 2-to-8 fold depending on the genetic position in the *fla/che* operon (Fig 7B). We conclude that although SwrB does not normally act to increase *fla/che* operon transcript levels, increased *fla/che* transcript levels nonetheless compensates for the absence of SwrB.

#### Class II–Deletions of the *codY* terminator

The *sob* class II alleles were identified by PCR amplification and directed sequencing of the *P*_*fla/che*_ promoter region. The PCR of some amplicons, however, were shorter than the wild type and sequencing indicated nested deletions such that the smallest common mutation deleted one arm of a Rho-independent terminator of the *codY* gene located immediately upstream of the *fla/che* operon (Fig 5B and Table 1). We note that similar deletions are thought to cause read-through transcription from the *codY* gene into the *fla/che* operon in previously identified suppressors of *swrA* (*soa*) [41], in 13 out of 23 newly-isolated of *soa* alleles (S2 Fig and Table 2), and also in suppressors of a dominant *degU32(Hy)* allele (*dhs*) [54]. When the *codY* terminator was deleted in the *sob37* genetic background, *fla/che* transcript levels increased from 4-to-14 fold depending on the genetic position in the *fla/che* operon (Fig 7C). We conclude that the class II *codY* terminator deletions compensated for the absence of SwrB in a manner similar to the class I alleles by increased expression of the *fla/che* operon transcript.

#### Class III–Mutations upstream of *P*_*fla/che*_

The *sob* class III alleles were identified by PCR amplification and directed sequencing of the *P*_*fla/che*_ promoter region. Four independently isolated *sob* alleles contained a single base pair mutation at position -110 and one *sob* allele contained a single base pair point mutation at position -104 relative to the *fla/che* transcriptional start site, upstream of the *P*_*fla/che*_ promoter (Fig 5B). Like the *sob* class I and class II mutations, the class III *sob6* allele elevated *fla/che* transcript levels, but to a lesser extent: only 1-to-4 fold depending on the position in the operon (Fig 7D). To test the role of the class III mutations on *P*_*fla/che*_ promoter activity, the *sob6* allele was cloned as part of the *P*_*fla/che*_ promoter region upstream of a promoter-less *lacZ* gene and inserted at an ectopic locus (*amyE*::*P*_*fla/che*_^*sobclassIII*^ *-lacZ*). Unlike the class I alleles, the class III allele did not increase *P*_*fla/che*_ reporter expression in the wild type, *swrB*, or *swrA* mutant backgrounds (Fig 6, compare white and black bars within strains). Thus, the class III alleles appeared to bypass the absence of SwrB without increasing transcription initiation from the *P*_*fla/che*_ promoter.

We considered the possibility that the *sob* class III alleles functioned *in trans* by mutating an as-yet undiscovered diffusible gene product encoded within the *P*_*fla/che*_ promoter region. The *sob6* allele did not appear to cause a loss-of-function phenotype to a diffusible gene product, however, because the *swrB* swarming motility defect was not restored to the *swrB sob6* background when the wild type *P*_*fla/che*_ promoter region was introduced at an ectopic site (S3A Fig). Furthermore, the *sob6* allele did not appear to cause a gain-of-function phenotype in a diffusible gene product because swarming motility was not rescued to a *swrB* mutant when the *P*_*fla/che*_^*sob6*^ promoter region was ectopically integrated (S3B Fig). We conclude that the *sob6* allele does not function in *trans*, but rather functions in *cis* in a manner seemingly independent of activating transcription initiation from the *P*_*fla/che*_ promoter. Similar mutations in the -110/-114 region were not in any of 23 newly-isolated suppressors of *swrA* (*soa*) and thus the region could potentially represent an enhanced SwrA binding site (S2 Fig and Table 2). Mutations in this region, however, do not act like SwrA to increase expression of the *P*_*fla/che*_ promoter and fall outside of the putative binding site of the response regulator DegU with which SwrA has been reported to interact [54,57–59]. What recognizes the -110/-114 element is unknown.

#### Class IV–Mutation of the intergenic region upstream of *cspB*

The four *sob* class IV alleles were not within the *P*_*fla/che*_ promoter region, and classical SPP1 transduction-based genetic linkage analysis failed due to an inability to generate linked transposon insertions. Instead, the *sob* class IV alleles were identified by Illumina whole-genome sequencing. Three of the class IV *sob* alleles were large deletions that overlapped with one another and all had in common the deletion of the gene *cspB*, encoding the RNA binding cold shock protein CspB, and the upstream intergenic region [60,61] (Fig 5C). Likewise, the fourth allele was a single base pair mutation in the intergenic region upstream of *cspB* and within the overlapping region common to the other three alleles. One way in which *sob* class IV alleles could restore motility to the *swrB* mutant is by increasing the expression of the *fla/che* operon in a manner similar to *sob* classes I, II, and III. The *swrB sob24* background containing a deletion spanning the intergenic region and *cspB* increased *fla/che* operon transcript abundance 2-to-7 fold but did not increase expression of the *P*_*fla/che*_ *-lacZ* reporter over wild type (Fig 7E and S4 Fig). We conclude that the class IV alleles rescued the *swrB* swarming defect by mutating a *trans* factor that directly or indirectly reduced *fla/che* operon transcript levels.

To explore the identity of the putative *trans* factor, four nested complementation constructs encoding combinations of *cspB*, the intergenic region, and the adjacent *yhcJ* gene were integrated at the ectopic *amyE* locus in the chromosome (Fig 5C). Only those constructs that contained both the intergenic region and the *cspB* open reading frame were able to restore swarming inhibition to the *swrB sob24* mutant (Fig 4H). Thus, one potentially relevant diffusible gene product disrupted by the *sob* class IV alleles was the CspB protein. To test the role of *cspB* in the inhibition of swarming motility in the *swrB* mutant, an in-frame markerless deletion was generated in the *cspB* open reading frame that left the upstream intergenic region intact. Deletion of *cspB* in an otherwise wild type background resulted in wild type swarming behavior but did not rescue swarming in the absence of SwrB (Fig 4I). We conclude that the *sob* class IV mutants rescued *swrB* swarming by mutation of a gene product emanating from the intergenic region upstream of *cspB*. The molecular nature of the diffusible gene product encoded upstream of the *cspB* gene is unknown but its mutation compensates for the absence of SwrB by increasing *fla/che* transcript levels like the *sob* class I, II, and III alleles.

#### Class V–Enhanced translation of FliP

The single *sob* class V allele (*sob22*) was found to be genetically linked to the *swrB*::*tet* parental allele by classical SPP1-transduction based linkage analysis. Sequencing of *fla/che* operon DNA upstream of *swrB* revealed a missense point mutation near the 3’ end of the *fliO* gene (FliO^K212E^) (Fig 5D and Table 1). One way in which the *sob22* mutation might restore swarming to the *swrB* mutant is by the generation of a gain-of-function substitution in FliO. To determine the consequence of the *sob22* allele on the function of FliO, the native *fliO* gene was first mutated by an in-frame marker-less deletion and then complemented with either the wild type *fliO* allele or the *fliO* ^*sob22*^ allele cloned downstream of the *P*_*fla/che*_ promoter region and inserted at the ectopic *amyE* locus (*amyE*::*P*_*fla/che*_ *-fliO* and *amyE*::*P*_*fla/che*_ *-fliO* ^*sob22*^). Deletion of *fliO* abolished swarming motility and the *fliO* deletion was partially complemented when either the wild type or *sob22* allele of *fliO* was ectopically expressed (Fig 4J). Neither the *fliO* wild type allele nor the *fliO* ^*sob22*^ allele, however, rescued swarming motility to a *swrB fliO* double mutant (Fig 4K). We conclude that the *fliO* ^*sob22*^ allele resulted in a neutral FliO^K212E^ substitution that did not suppress the *swrB* phenotype.

Another way in which the *sob22* mutation might restore swarming to the *swrB* mutant is by enhancing translation of the *fliP* gene (Fig 5D). The open reading frame of *fliP* is immediately downstream of, and overlaps with, the open reading frame of *fliO*. The *sob22* allele is an A to G transition fifteen base pairs upstream of the *fliP* translational start and results in a sequence with increased similarity to the consensus for a Shine-Dalgarno ribosome binding site (AGAAGA > AGGAGA) [62] (Fig 5D). To determine the consequence of the *sob22* allele on FliP, the native *fliP* gene was first mutated by an in-frame markerless deletion and then complemented with either the wild type *fliP* allele or the *fliP* ^*sob22*^ allele cloned downstream of the *P*_*fla/che*_ promoter region and inserted at the ectopic *amyE* locus (*amyE*::*P*_*fla/che*_ *-fliP* and *amyE*::*P*_*fla/che*_ *-fliP* ^*sob22*^). Deletion of *fliP* abolished swarming motility and the *fliP* deletion was partially complemented with the wild type *fliP* allele and fully complemented by the *fliP* ^*sob22*^ allele (Fig 4L). Whereas the wild type allele of *fliP* did not restore swarming motility to a *swrB fliP* double mutant, the *fliP* ^*sob22*^ allele rescued swarming to a level comparable to that observed in the original *sob22* background (Fig 4M). We conclude that *sob22* suppresses the *swrB* non-swarming phenotype by improving the translation of FliP.

To determine whether increasing expression of FliP by another mechanism was sufficient to restore swarming motility to the *swrB* mutant, the wild type *fliP* allele was cloned downstream of the IPTG-inducible *P*_*hyspank*_ promoter and inserted at the *amyE* locus (*amyE*::*P*_*hsypank*_ *-fliP*). As specificity controls, the *fliO* and *fliQ* genes encoded immediately upstream and downstream of *fliP* respectively were separately cloned under the *P*_*hyspank*_ promoter and inserted at the *amyE* locus. Whereas IPTG induction of *fliP* restored swarming motility to the *swrB* mutant, IPTG induction of *fliO* (Fig 4N) and *fliQ* did not (S5 Fig). FliO, FliP and FliQ are core transmembrane components of the flagellar type III secretion export apparatus, and we conclude that increased synthesis of FliP specifically is sufficient to bypass the requirement of SwrB in swarming motility. We infer that increased expression of FliP likely accounts for the Class I, II, III, and IV mutations that act to increase the expression of the *fla/che* operon, of which *fliP* is a member (Fig 5A).

#### Class VI–Gain-of-function allele in FliG

The two *sob* class VI alleles were independently-isolated and identified using Illumina whole genome sequencing. Both *sob* class VI alleles were identical missense mutations in the *fliG* gene encoding the flagellar rotor protein FliG (FliG^Q132R^) (Fig 5E and Table 1). To determine the consequences of FliG^Q132R^, the native *fliG* gene was first mutated by an in-frame markerless deletion and complemented with the either the wild type *fliG* or the *fliG* ^*Q132R*^ allele cloned downstream of the *P*_*fla/che*_ promoter region and inserted at the ectopic *amyE* locus (*amyE*::*P*_*fla/che*_ *-fliG* and *amyE*::*P*_*fla/che*_ *-fliG* ^*Q132R*^). Deletion of *fliG* abolished swarming motility and the *fliG* deletion was partially complemented by the wild type FliG protein (Fig 4O). In contrast, the FliG^Q132R^ protein fully complemented the swarming defect of the *fliG* mutant and thus appeared to have enhanced activity (Fig 4O). Furthermore, the swarming defect of a *swrB fliG* double mutant was rescued in the presence of FliG^Q132R^ but not wild type FliG (Fig 4P). We conclude that the FliG^Q132R^ substitution confers a gain-of-function phenotype sufficient to restore swarming in the absence of SwrB.

**Fig 8:**
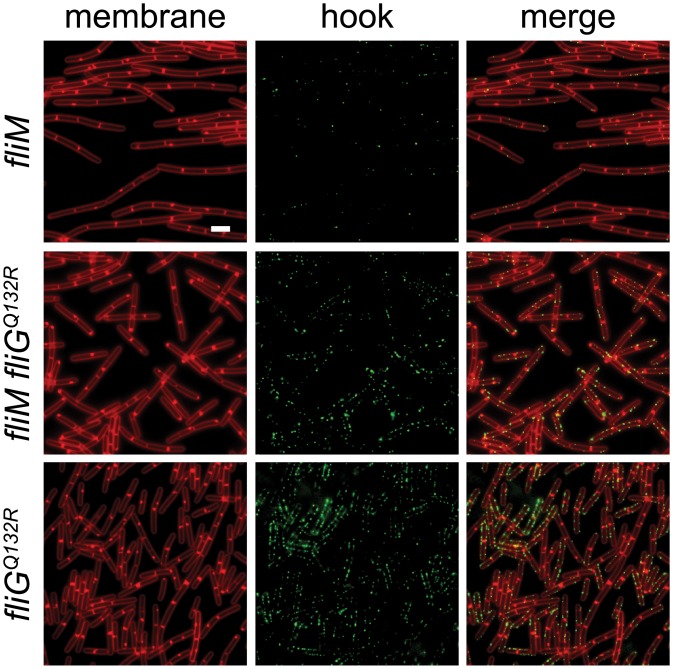
FliG^Q132R^ rescues hook assembly to a FliM-deficient background. Fluorescence micrographs of the indicated genotypes stained for membranes (FM 4–64, false-colored red) and for the FlgE^T123C^ hook variant (Alexa fluor 488 maleimide, false-colored green). The following strains were used to generate this panel: *fliM* (DK1564), *fliM fliG* ^*Q132R*^ (DK1476), *fliG* ^*Q132R*^ (DK1475). Scale bar is 4 μm.

The FliG^Q132R^ substitution is located in the central armadillo (ARM) motif associated with polymerization of the rotor and assembly of the FliM C-ring component [32,63,64]. In *E*. *coli* and *S*. *enterica*, loss-of-function mutations in either the FliG ARM motif or FliM result in assembly defects of extracellular flagellar components [31,34–37,63]. Consistent with a role for FliM in flagellar assembly, mutation of *fliM* in *B*. *subtilis* reduced the flagellar hook frequency similar to that observed in the absence of SwrB (Fig 8). Introduction of the gain-of-function *fliG* ^*Q132R*^ allele to the *fliM* mutant background, however, increased hook frequency similar to that found in the isogenic *fliG* ^*Q132R*^ allele alone (Fig 8). We conclude that the gain-of-function *fliG* ^*Q132R*^ allele bypasses the absence of FliM and increases the frequency of hook assembly. We infer that FliG^Q132R^ alters the conformation of the flagellar rotor and that this conformation is normally adopted only after complete assembly of FliM into the C-ring.

### SwrB activates hook secretion in a manner dependent on FliF and FliP

**Fig 9:**
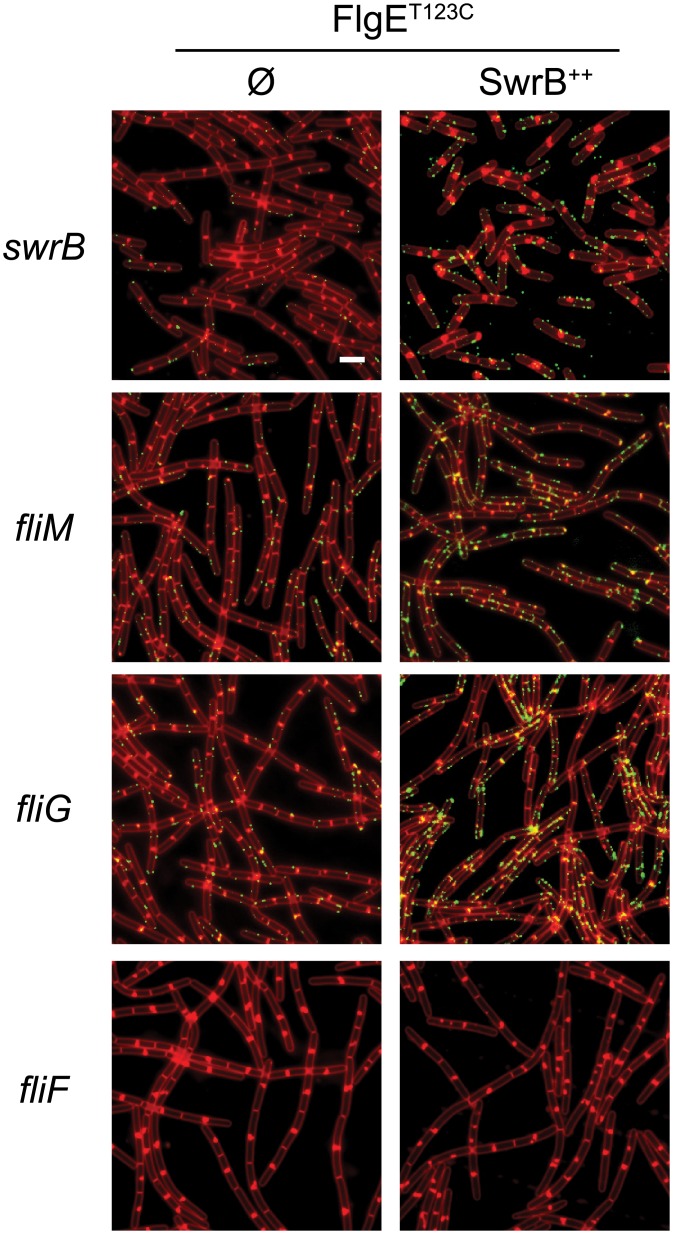
C-ring mutants phenocopy SwrB mutants for hook synthesis and SwrB overexpression bypasses C-ring defects. Fluorescence micrographs of the flagellar hook (FlgE^T123C^) in the indicated genetic backgrounds. Ø column indicates no additional genetic modifications whereas (SwrB^++^) indicates the presence of an ectopically integrated *P*_*hyspank*_ *-swrB* construct induced with 1 mM IPTG. Membranes stained with FM4-64 and false colored red. Hooks stained with maleimide alexa fluor 488 and false colored green. The following strains were used to generate this panel: *swrB* (DK478), *swrB* ^++^ (DK2089), *fliM* (DK1564), *fliM* SwrB^++^ (DK2087), *fliG* (DK2098), *fliG* SwrB^++^ (DK3041), *fliF* (DK3135), and *fliF* SwrB^++^ (DK3137). Scale bar is 4 μm.

Mutations in FliP and FliG that suppressed the absence of SwrB suggested that the mechanism by which SwrB enhanced hook assembly was related to flagellar secretion by way of basal body structure. Therefore, we further explored the relationship of SwrB to components of the basal body and C-ring for enhancing hook polymerization. To do so, strains were generated that contained the FlgE^T123C^ allele for fluorescent labeling of the flagellar hook in backgrounds mutated for SwrB, FliM, FliG, and FliF. Cells mutated for either FliM or FliG displayed a low frequency of flagellar hooks and resembled cells mutated for SwrB (Fig 9). By contrast, no hooks were detected in cells mutated for FliF (Fig 9). Next, the *swrB* gene was cloned downstream of an IPTG inducible *P*_*hyspank*_ promoter and integrated at an ectopic site in each of the strains tested (*amyE*::*P*_*hyspank*_ *-swrB*). In the presence of IPTG, overexpression of SwrB increased the frequency of hooks for the *swrB*, *fliM* and *fliG* mutants but did not restore hook formation to the *fliF* mutant (Fig 9). We conclude that the activation of hook assembly by SwrB does not require FliG or FliM, but that FliG^Q132R^ nonetheless compensates for the absence of SwrB. We further conclude that the basal body structural component FliF is required for SwrB to activate hook assembly.

**Fig 10:**
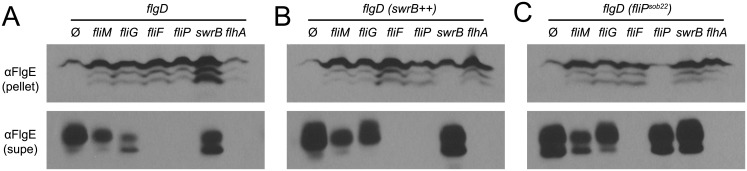
SwrB overexpression bypasses the hook protein secretion hook in cells mutated for FliM and FliG but not cells mutated for FliF. A) FlgE secretion assay in a hook cap (*flgD*) mutant background. Mutation of the hook cap FlgD has previously been shown to abolish FlgE polymerization and allow constitutive secretion of FlgE into the extracellular environment [51,65]. Cell pellet (pellet) and cell supernatant (supe) samples were harvested from *B*. *subtilis* cultures of the indicated genetic background and probed with anti-FlgE primary antibody. The following strains were used to generate the panel: *ΔflgD* (DK2102), *ΔflgD ΔfliM* (DK2103), *ΔflgD ΔfliG* (DK2201), *ΔflgD ΔfliF* (DS2229), *ΔflgD ΔfliP* (DK2199), *ΔflgD ΔswrB* (DK2214), and *ΔflgD ΔflhA* (DK2198). B) FlgE secretion assay in a hook cap (*flgD*) mutant background and in which *swrB* is artificially expressed from the IPTG-inducible *P*_*hyspank*_ promoter (*swrB* ^*++*^). Strains contain the *thrC*::*P*_*hyspank*_ *-swrB* construct and were grown in the presence of 1 mM IPTG. The following strains were used to generate the panel: *ΔflgD swrB* ^*++*^ (DK2282), *ΔflgD ΔfliM swrB* ^*++*^ (DK2283), *ΔflgD ΔfliG swrB* ^*++*^ (DK2284), *ΔflgD ΔfliF swrB* ^*++*^ (DK2285), *ΔflgD ΔfliP swrB* ^*++*^ (DK2286), *ΔflgD ΔswrB swrB* ^*++*^ (DK2287), and *ΔflgD ΔflhA swrB* ^*++*^ (DK2288). C) FlgE secretion assay in a hook cap (*flgD*) mutant background and in which the *fliP* ^*sob22*^ allele was ectopically expressed from the native *fla/che* promoter. The following strains were used to generate the panel: *ΔflgD fliP* ^*sob22*^ (DK2289), *ΔflgD ΔfliM fliP* ^*sob22*^ (DK2290), *ΔflgD ΔfliG fliP* ^*sob22*^ (DK2291), *ΔflgD ΔfliF fliP* ^*sob22*^ (DK2292), *ΔflgD ΔfliP fliP* ^*sob22*^ (DK2293), *ΔflgD ΔswrB fliP* ^*sob22*^ (DK2294), *ΔflgD ΔflhA fliP* ^*sob22*^ (DK2295).

FliF could be required for SwrB activation of hook assembly because FliF also serves as the polymerization platform for the flagellar rod and hook. Thus, SwrB could stimulate flagellar secretion in the absence of FliF but the hook subunits would accumulate in the supernatant due to an inability to polymerize. To determine the effect SwrB has on hook protein secretion, strains were generated that were mutated for the extracellular hook chaperone protein FlgD such that all strains would fail to polymerize FlgE, and FlgE would thus be secreted into the supernatant [51,65]. Cells mutated for FliM and FliG reduced the amount of secreted hook protein whereas cells mutated for FliF, FliP, and FlhA abolished hook secretion (Fig 10A). Next, the IPTG-inducible *P*_*hyspank*_ *-swrB* construct was added to each strain to determine the effect that over-expression of SwrB would have on hook secretion. When SwrB was over-expressed, the amount of hook protein in the supernatant appeared to increase in the *fliM*, *fliG*, *and swrB* mutants, but not in the *fliF*, *fliP*, or *flhA* mutants (Fig 10B). Finally, addition of the *P*_*fla/che*_ *-fliP* ^*sob22*^ complementation construct provided *in trans* appeared to increase FlgE secretion in *fliM*, *fliG*, *swrB*, and *fliP* mutants but not in the *fliF* or *flhA* mutant (Fig 10C). We conclude FliF, FliP, and FlhA are downstream of SwrB for hook protein secretion. In sum, we conclude that SwrB activates an early flagellar morphogenetic checkpoint by catalyzing a FliF-basal body conformation that activates the type III secretion export apparatus for hook subunit secretion.

## Discussion

Flagellar regulation is morphogenetically coupled to flagellar structural intermediates to ensure that the structural subunits are assembled in the proper order. A classic example of morphogenetic coupling is illustrated by how the completion of the flagellar hook induces a change in the substrate specificity of the export apparatus and governs the expression of the flagellar filament protein via secretion of the anti-sigma factor FlgM [2,27]. Here we find evidence of an earlier morphogenetic coupling event in which SwrB and the completion of the flagellar basal body govern the secretion of the hook (and likely rod) structural subunits. Given that the type III secretion export apparatus appears to be the first flagellar subdomain assembled within the membrane, we suggest that if the export apparatus was immediately active upon assembly, structural components would be secreted in the absence of the basal body upon which they are polymerized. Instead, it appears that the export apparatus becomes functional only after the basal body is complete; an event indicated by a conformational change adopted by the flagellar base plate protein FliF. Thus, basal body completion is a discrete flagellar morphogenetic checkpoint and we argue that the *B*. *subtilis* membrane protein SwrB potentiates the ability of the basal body to adopt a “completed” conformation.

SwrB (YlxL) was originally discovered as the product encoded by the last gene in the 32 gene *fla/che* operon and was shown to be required for the activation of σ^D^-dependent gene expression and swarming motility [38,39,41]. Here we account for both previously reported phenotypes of the *swrB* mutant. We found that cells defective for SwrB were unable to swarm because they failed to synthesize wild type numbers of flagellar filaments and resembled the hypoflagellated state of cells defective for another swarming regulator, SwrA [53]. The SwrB defect in flagellar number was not due to a reduction in the number of flagellar basal bodies as seen in cells defective for SwrA however, but rather due to a reduction in the number of flagellar hooks. The *swrB* mutant defect in hook synthesis is consistent with the observed reduction in σ^D^-dependent gene expression as hook completion is needed to antagonize the σ^D^ anti-sigma factor FlgM [39,51]. Thus, SwrB increased the probability that basal bodies became proficient for hook assembly. Spontaneous suppressors that restored swarming motility to *swrB* mutants indicated that SwrB increased the frequency of hook assembly by activating hook subunit secretion via the type III secretion component FliP.

FliP is a transmembrane protein that is incorporated early in the nascent basal body and required for nucleation of other components of the flagellar type III secretion export apparatus FliO, FliQ, FliR, FlhA and FlhB [5,66,67]. Mutation of FliP abolishes secretion, and while the precise function of FliP is unknown, it appears to be regulated by at least two mechanisms. First, the FliP N-terminal signal sequence appears to be inhibitory in *S*. *enterica* and is processed presumably after assembly of the export apparatus [5,68]. *B*. *subtilis* FliP however lacks the N-terminal signal sequence altogether, perhaps an indication of the need for additional regulatory mechanisms [68,69]. Second, the levels of FliP appear to be important in *S*. *enterica* as the accessory protein FliO protects FliP from proteolytic degradation [21,22]. Here we further support the importance of FliP protein levels in *B*. *subtilis* as 22 out of 24 spontaneous suppressor-of-*swrB* (*sob*) mutations restored swarming to a *swrB* mutant by increasing FliP expression.

FliP was directly implicated by a single suppressor-of-*swrB* (*sob*) mutation that mutated the FliP Shine-Dalgarno sequence closer to consensus, thus enhancing translation. Furthermore, enhanced transcription of the native *fliP* gene within the *fla/che* operon by another 21 *sob* alleles or an ectopically integrated *fliP* gene expressed from artificial IPTG-inducible promoter was sufficient to rescue swarming to a *swrB* mutant. FliP is hypothesized to sit within the confines of a ring of the basal body protein FliF, the stoichiometry of which along with the rest of the type III secretion export apparatus components is thought to be definite and precise [5]. Thus, how extra copies of wild type FliP would improve swarming and/or become incorporated into basal bodies is unclear. Perhaps extra FliP protein titrates an inhibitor of the type III secretion export apparatus. Alternatively, FliP is thought to be one of the earliest proteins assembled in the flagellum and overexpression may increase the population of FliP molecules in a secretion-active conformation that preferentially promotes basal body nucleation. A potential candidate for either the inhibitor and/or conformational regulator of the type III secretion system is found in the final class of SwrB suppressors that fall within the rotor protein FliG.

FliG forms the gear-like rotor that docks to the cytoplasmic surface of the FliF basal body protein, interacts with the MotA/MotB proton channel stator, and serves as a scaffold for the assembly of the FliM/FliN(FliY) cytoplasmic C-ring [33,70–75]. Although FliG, FliM, and FliN are found in the cytoplasm, it has long been known that mutations in each protein cause extracytoplasmic flagellar assembly defects [31–37]. Here we show that a gain-of-function mutation found in the ARM motif that controls FliG polymerization bypasses the C-ring requirement for flagellar hook (but not flagellar filament) assembly [64] (Fig 8 and S6 Fig). We posit that proper conformation of the FliG rotor *in vivo*, normally promoted by the completion of the C-ring and the presence of SwrB, activates the type III secretion export apparatus. Since FliG and the export apparatus are not known to directly interact, we infer that a regulatory conformational change is propagated from the rotor through basal body protein FliF [74].

FliF is a critical transmembrane structural protein that defines the flagellar basal body as it docks to the FliG rotor, surrounds the type III export apparatus, and forms a polymerization platform for the rod [4,76–78]. FliF has been hypothesized to exist in active and inactive conformations. In *S*. *enterica*, FliF has a large periplasmic domain that may regulate the export apparatus as electron microscopy of FliF rings show two conformations, one with a lumen that appears to be open and one in which the lumen appears to be closed [4,79–81]. Furthermore, a FliF closed conformation was hypothesized to be adopted when the poorly-understood periplasmic protein FliE was mutated [6,82,83]. In *B*. *subtilis*, FliF is required for secretion as cells defective in FliF fail to secrete both the early class substrate FlgE and the late class substrate FlgM (Fig 10) [52]. Thus, FliF could be required for secretion by surrounding the type III export apparatus and regulating its function.

**Fig 11:**
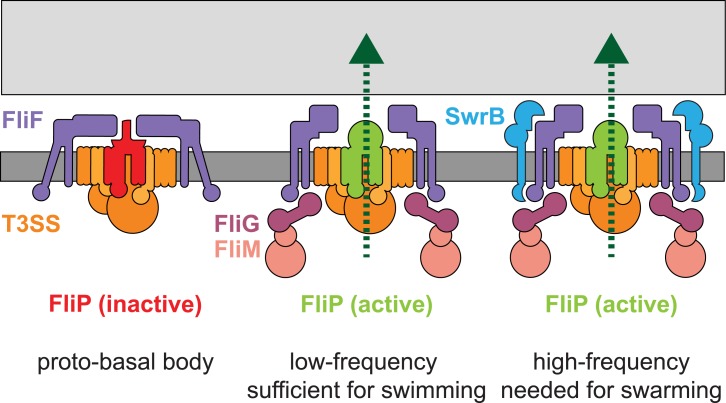
SwrB activates flagellar type III secretion. A model depicting the early formation of a secretion inactive proto-basal body (left). Basal bodies spontaneously mature to an active state at a low frequency in the absence of SwrB and the flagella synthesized are sufficient to swim through liquid but insufficient to swarm over surfaces (middle). SwrB is required to accelerate the basal body maturation process, increase the number of secretion active basal bodies, increase the number of flagellar hooks and therefore increase flagellar number to levels needed for swarming. We hypothesize that a conformational change in FliF is mediating SwrB activation, because SwrB requires FliF, FliF is required for flagellar secretion, FliF occupies a space between FliG, FliP and SwrB, and FliF complexes have been reported to adopt open and closed conformations. Dark gray bar indicates membrane, light gray bar indicates peptidoglycan, purple shapes indicate FliF, magenta shapes indicate FliG, pink shapes indicate FliM and FliN/Y, orange indicates the secretion complex, and light blue indicates SwrB. FliP is colored red to indicate an inactive secretion complex or light green to indicate an active secretion complex. Protein secretion is indicated by a dark green dotted line.

Based on cytological and genetic suppressor data, we conclude that SwrB functions as an assembly chaperone to enhance the probability that the flagellar basal body adopts a conformation proficient for secretion (Fig 11). We propose that the flagellar type III secretion apparatus and FliF form first in the membrane as a “proto-basal body” that is inactive for export (Fig 11A) [7]. The proto-basal body is able to spontaneously mature to become proficient for hook secretion at a low frequency (Fig 11B). Under normal conditions, the frequency of proto-basal body maturation is increased by both SwrB and assembly of the C-ring as the absence of either share a low hook-to-basal body ratio (Fig 11C). Indeed, SwrB and FliG appear to be required at the same step as FliG gain-of-function alleles that enhance rotor stability bypass the requirement of SwrB for hook assembly, and artificial overexpression of SwrB bypasses the need for FliG. The most likely convergence point for SwrB and FliG is the membrane-basal body protein FliF, as FliG is a known interactor and SwrB, itself a membrane protein, could be adjacent. We hypothesize that the previously documented changes in FliF conformation are responsible for activating the export apparatus and do so by activating the membrane protein FliP. In sum, maturation of the flagellar basal body to a secretion proficient state is a morphogenetic checkpoint that must be passed prior to proceeding to the export and assembly of more distal components.

SwrB is conserved only in members of the genus *Bacillus* but the notion that flagellar basal body completion acts as a conformational liscencing event to permit subsequent flagellar secretion is consistent with genetic and biochemical observations in a variety of systems. In *Campylobacter jejuni*, the conformation adopted by the completed basal body activates expression of the rod and hook genes as a separate regulatory “tier”, and similar “4-tier” flagellar regulatory hierarchies have been demonstrated in *Caulobacter crescentus* and *Pseudomonas aeruginosa* [84–91]. Thus instead of regulating the type III export apparatus functionally, completion of the basal body transcriptionally controls the availability of the rod and hook cargo. Between our results in *B*. *subtilis* and the “4-tier” flagellar systems, we suggest that the rod should be considered separate from the basal body and therefore the structural domains of the flagellum should be considered the “basal body”, the “rod-hook”, and the “filament”, at least for these organisms. Finally, our conformation control model is consistent with the notion that a conformational change propagates from the needle tip to the export apparatus at the base of the type III secretion injectosomes of pathogens to activate the secretion of effectors in response to direct contact with host cells [92–94]. Thus, we conclude that the activation of secretion is a regulated morphogenetic checkpoint, and disrupting functional regulators could be a strategy to attenuate both flagella and injectisome virulence factors.

## Materials and Methods

### Strains and growth conditions

*B*. *subtilis* strains were grown in Luria-Bertani (LB) (10 g tryptone, 5 g yeast extract, 5 g NaCl per L) broth or on LB plates fortified with 1.5% Bacto agar at 37°C. When appropriate, antibiotics were included at the following concentrations: 10 μg/ml tetracycline, 100 μg/ml spectinomycin, 5 μg/ml chloramphenicol, 5 μg/ml kanamycin, and 1 μg/ml erythromycin plus 25 μg/ml lincomycin (*mls*). Isopropyl β-D-thiogalactopyranoside (IPTG, Sigma) was added to the medium at the indicated concentration when appropriate. For the swarm expansion assay, swarm agar plates containing 25 ml LB fortified with 0.7% Bacto agar were prepared fresh and the following day were dried for a total of 20 minutes in a laminar flow hood (see below).

### Strain construction

All constructs were first introduced into the competent ancestral strain DS2569 by natural competence and then transferred to the 3610 background using SPP1-mediated generalized phage transduction or by transformation in the competent ancestral strain DK1042 (as indicated by the presence of the *comI* ^*Q12L*^ allele in the genotype) [95,96]. All strains used in this study are listed in Table 3. All plasmids used in this study are listed in S1 Table. All primers used in this study are listed in S2 Table.

**Table 3:** Strains^a^.

Strain	Genotype
3610	Wild type
DK52	*ΔfliO amyE*::*P*_*flache*_ *-fliO spec*
DK53	*ΔfliO amyE*::*P*_*flache*_ *-fliO* ^*sob22*^ *spec*
DK54	*swrB*::*tet ΔfliO*
DK65	*swrB*::*tet ΔfliO amyE*::*P*_*flache*_ *-fliO spec*
DK66	*swrB*::*tet ΔfliO amyE*::*P*_*flache*_ *-fliO* ^*sob22*^ *spec*
DK284	*ΔswrA thrC*::*P*_*flache*_ *–lacZ mls*
DK285	*ΔswrB thrC*::*P*_*flache*_ *–lacZ mls*
DK288	*ΔswrA lacA*::*P*_*hag*_ *-lacZ mls*
DK289	*ΔswrB lacA*::*P*_*hag*_ *-lacZ mls*
DK313	*flgM*::*tet lacA*::*P*_*hag*_ *-lacZ mls*
DK318	*ΔswrA flgM*::*tet lacA*::*P*_*hag*_ *-lacZ mls*
DK319	*ΔswrB flgM*::*tet lacA*::*P*_*hag*_ *-lacZ mls*
DK478	*ΔflgE swrB*::*tet amyE*::*P*_*flache*_ *-flgE* ^*T123C*^ *cat*
DK479	*ΔfliM swrB*::*tet amyE*::*P*_*flache*_ *-fliM-gfp spec*
DK480	*ΔflgE swrA*::*kan amyE*::*P*_*flache*_ *-flgE* ^*T123C*^ *cat*
DK486	*flgM*::*tet amyE*::*P*_*hag*_ *-hag* ^*T209C*^ *spec*
DK487	*ΔswrA flgM*::*tet amyE*::*P*_*hag*_ *-hag* ^*T209C*^ *spec*
DK488	*ΔswrB flgM*::*tet amyE*::*P*_*hag*_ *-hag* ^*T209C*^ *spec*
DK519	*swrB*::*tet ΔfliP*
DK568	*ΔfliG amyE*::*P*_*flache*_ *-fliG* ^*sob28*^ *spec*
DK572	*swrB*::*tet ΔfliP amyE*::*P*_*flache*_ *-fliP spec*
DK574	*swrB*::*tet ΔfliP amyE*::*P*_*flache*_ *-fliP* ^*sob22*^ *spec*
DK577	*ΔfliP amyE*::*P*_*flache*_ *-fliP spec*
DK579	*ΔfliP amyE*::*P*_*flache*_ *-fliP* ^*sob22*^ *spec*
DK584	*ΔfliG swrB*::*tet*
DK598	*ΔfliG swrB*::*tet amyE*::*P*_*flache*_ *-fliG* ^*sob28*^ *spec*
DK601	*swrB*::*tet amyE*::*P*_*hyspank*_ *-fliP spec*
DK737	*ΔfliG amyE*::*P*_*flache*_ *-fliG spec*
DK738	*ΔfliG swrB*::*tet amyE*::*P*_*flache*_ *-fliG spec*
DK1042	*comI* ^*Q12L*^ [96]
DK1056	*swrA*::*tet soa5*
DK1057	*swrA*::*tet soa6*
DK1058	*swrA*::*tet soa7*
DK1059	*swrA*::*tet soa8*
DK1060	*swrA*::*tet soa9*
DK1062	*swrA*::*tet soa11*
DK1063	*swrA*::*tet soa12*
DK1064	*swrA*::*tet soa13*
DK1065	*swrA*::*tet soa14*
DK1066	*swrA*::*tet soa15*
DK1067	*swrA*::*tet soa16*
DK1068	*swrA*::*tet soa17*
DK1069	*swrA*::*tet soa18*
DK1070	*swrA*::*tet soa19*
DK1071	*swrA*::*tet soa20*
DK1072	*swrA*::*tet soa21*
DK1073	*swrA*::*tet soa22*
DK1074	*swrA*::*tet soa23*
DK1075	*swrA*::*tet soa24*
DK1076	*swrA*::*tet soa25*
DK1077	*swrA*::*tet soa26*
DK1078	*swrA*::*tet soa27*
DK1079	*swrA*::*tet soa28*
DK1116	*ΔswrA amyE*::*P*_*flache*_^*sob6*^ *–lacZ spec*
DK1117	*ΔswrB amyE*::*P*_*flache*_^*sob6*^ *–lacZ spec*
DK1118	*amyE*::*spec*
DK1129	*ΔswrA amyE*::*P*_*flache*_^*sob21*^ *–lacZ cat*
DK1130	*ΔswrB amyE*::*P*_*flache*_^*sob21*^ *–lacZ cat*
DK1230	*swrB*::*tet amyE*::*P*_*hyspank*_ *-fliO spec*
DK1285	*fliG* ^*Q132R*^ *comI* ^*Q12L*^
DK1412	*fliG* ^*Q132R*^ *ΔfliM comI* ^*Q12L*^
DK1475	*fliG* ^*Q132R*^ *ΔflgE amyE*::*P*_*flache*_ *-flgE* ^*T123C*^ *cat comI* ^*Q12L*^
DK1476	*fliG* ^*Q132R*^ *ΔfliM ΔflgE amyE*::*P*_*flache*_ *-flgE* ^*T123C*^ *cat comI* ^*Q12L*^
DK1564	*ΔflgE ΔfliM amyE*::*P*_*flache*_ *-flgE* ^*T123C*^ *cat*
DK1905	*ΔcspB comI* ^*Q12L*^
DK1937	*ΔcspB swrB*::*tet*
DK2015	*swrB*::*tet sob24 amyE*::*sob24comp1 spec*
DK2016	*swrB*::*tet sob24 amyE*::*sob24comp3 spec*
DK2017	*swrB*::*tet sob24 amyE*::*sob24comp2 spec*
DK2018	*swrB*::*tet sob24 amyE*::*sob24comp4 spec*
DK2087	*ΔflgE ΔfliM amyE*::*P*_*flache*_ *-flgE* ^*T123C*^ *cat thrC*::*P*_*hyspank*_ *-swrB mls comI* ^*Q12L*^
DK2089	*ΔflgE swrB*::*tet amyE*::*P*_*flache*_ *-flgE* ^*T123C*^ *cat thrC*::*P*_*hyspank*_ *-swrB mls comI* ^*Q12L*^
DK2098	*ΔflgE ΔfliG amyE*::*P*_*flache*_ *-flgE* ^*T123C*^ *cat comI* ^*Q12L*^
DK2102	*ΔflgD comI* ^*Q12L*^
DK2103	*ΔflgD ΔfliM comI* ^*Q12L*^
DK2198	*ΔflgD ΔflhA comI* ^*Q12L*^
DK2199	*ΔflgD ΔfliP comI* ^*Q12L*^
DK2201	*ΔflgD ΔfliG comI* ^*Q12L*^
DK2214	*ΔflgD ΔswrB*
DK2229	*ΔflgD ΔfliF*
DK2282	*ΔflgD thrC*::*P*_*hyspank*_ *-swrB mls comI* ^*Q12L*^
DK2283	*ΔflgD ΔfliM thrC*::*P*_*hyspank*_ *-swrB mls comI* ^*Q12L*^
DK2284	*ΔflgD ΔfliG thrC*::*P*_*hyspank*_ *-swrB mls comI* ^*Q12L*^
DK2285	*ΔflgD ΔfliF thrC*::*P*_*hyspank*_ *-swrB mls*
DK2286	*ΔflgD ΔfliP thrC*::*P*_*hyspank*_ *-swrB mls comI* ^*Q12L*^
DK2287	*ΔflgD ΔswrB thrC*::*P*_*hyspank*_ *-swrB mls*
DK2288	*ΔflgD ΔflhA thrC*::*Phsypank-swrB mls comI* ^*Q12L*^
DK2289	*ΔflgD thrC*::*P*_*fla/che*_ *-fliP* ^*sob22*^ *mls comI* ^*Q12L*^
DK2290	*ΔflgD ΔfliM thrC*::*P*_*fla/che*_ *-fliP* ^*sob22*^ *mls comI* ^*Q12L*^
DK2291	*ΔflgD ΔfliG thrC*::*P*_*fla/che*_ *-fliP* ^*sob22*^ *mls comI* ^*Q12L*^
DK2292	*ΔflgD ΔfliF thrC*::*P*_*fla/che*_ *-fliP* ^*sob22*^ *mls*
DK2293	*ΔflgD ΔfliP thrC*::*P*_*fla/che*_ *-fliP* ^*sob22*^ *mls comI* ^*Q12L*^
DK2294	*ΔflgD ΔswrB thrC*::*P*_*fla/che*_ *-fliP* ^*sob22*^ *mls*
DK2295	*ΔflgD ΔflhA thrC*::*P*_*fla/che*_ *-fliP* ^*sob22*^ *mls comI* ^*Q12L*^
DK3041	*ΔflgE ΔfliG amyE*::*P*_*flache*_ *-flgE* ^*T123C*^ *cat thrC*::*P*_*hyspank*_ *-swrB mls comI* ^*Q12L*^
DK3135	*ΔflgE ΔfliF amyE*::*P*_*flache*_ *-flgE* ^*T123C*^ *cat comI* ^*Q12L*^
DK3137	*ΔflgE ΔfliF amyE*::*P*_*flache*_ *-flgE* ^*T123C*^ *cat thrC*::*P*_*hyspank*_ *-swrB mls comI* ^*Q12L*^
DK3350	*ΔfliQ amyE*::*P*_*hyspank*_ *-fliQ spec*
DK3354	*ΔswrB amyE*::*P*_*hyspank*_ *-fliQ spec*
DS234	*swrB*::*tet* [38]
DS725	*swrA*::*tet soa3* [41]
DS726	*swrA*::*tet soa4* [41]
DS793	*amyE*::*P*_*flache*_ *-lacZ cat* [41]
DS908	*amyE*::*P*_*hag*_ *-gfp cat* [41]
DS1426	*amyE*::*P*_*flache*_^*sob21*^ *–lacZ cat* ^*b*^
DS1916	*amyE*::*P*_*hag*_ *-hag* ^*T209C*^ *spec* [43]
DS2415	*ΔswrA* [98]
DS2569	*ΔpBS32* [96]
DS4015	*ΔswrA amyE*::*P*_*hag*_ *-gfp cat* [49]
DS4034	*ΔswrA flgM*::*tet amyE*::*P*_*hag*_ *-gfp cat* [49]
DS4040	*ΔswrB amyE*::*P*_*hag*_ *-gfp cat*
DS4090	*ΔswrB flgM*::*tet amyE*::*P*_*hag*_ *-gfp cat*
DS4264	*flgM*::*tet amyE*::*P*_*hag*_ *-gfp cat*
DS4633	*swrB*::*tet sob4*
DS6468	*ΔfliO* [52]
DS6775	*ΔfliM* [53]
DS7063	*swrB*::*Tn10 spec sob6*
DS7064	*swrB*::*tet sob24*
DS7119	*ΔfliP* [52]
DS7357	*ΔfliG* [102]
DS7673	*ΔflgE amyE*::*P*_*flache*_ *-flgE* ^*T123C*^ *cat* [51]
DS8521	*ΔfliM amyE*::*P*_*fla/che*_ *-fliM-gfp spec* [53]
DS8600	*ΔswrA ΔfliM amyE*::*P*_*flache*_ *-fliM-gfp spec* [53]
DS9120	*amyE*::*P*_*flache*_^*sob6*^ *–lacZ spec*
DS9147	*swrB*::*tet sob20*
DS9148	*swrB*::*tet sob21*
DS9149	*swrB*::*tet sob22*
DS9150	*swrB*::*tet sob23*
DS9151	*swrB*::*tet sob24*
DS9152	*swrB*::*tet sob25*
DS9153	*swrB*::*tet sob26*
DS9154	*swrB*::*tet sob27*
DS9155	*swrB*::*tet sob28*
DS9156	*swrB*::*tet sob29*
DS9157	*swrB*::*tet sob30*
DS9158	*swrB*::*tet sob31*
DS9319	*ΔswrB amyE*::*P*_*hag*_ *-hag* ^*T209C*^ *spec*
DS9461	*lacA*::*P*_*hag*_ *-lacZ mls*
DS9515	*ΔswrA amyE*::*P*_*hag*_ *-hag* ^*T209C*^ *spec*
DS9816	*swrB*::*tet sob33*
DS9817	*swrB*::*tet sob34*
DS9818	*swrB*::*tet sob35*
DS9819	*swrB*::*tet sob36*
DS9831	*swrB*::*tet sob37*
DS9845	*swrB*::*tet sob38*
DS9846	*swrB*::*tet sob39*

^a^All strains are in the undomesticated 3610 genetic background unless otherwise indicated.

^b^DS1426 was previously published as *amyE*::*P*_*flache*_^*soa*^ *-lacZ cat* [37].

#### LacZ reporter constructs

To generate the β-galactosidase (*lacZ*) reporter construct pAP50, a PCR product containing the P_*flache*_ promoter including the *sob6* point mutation was amplified from *B*. *subtilis* strain DS7063 using primer pair 321/322. The PCR product was digested with EcoRI and BamHI and cloned into the EcoRI and BamHI sites of plasmid pDG1728, which carries a spectromycin-resistance marker and a polylinker upstream of the *lacZ* gene between two arms of the *amyE* gene [97].

#### In-frame deletions

To generate the *ΔfliO* in frame marker-less deletion construct, the region upstream of *fliO* was PCR amplified using the primer pair 1692/1693 and digested with EcoRI and XhoI, and the region downstream of *fliO* was PCR amplified using the primer pair 1694/1695 and digested with XhoI and BamHI. The two fragments were then simultaneously ligated into the EcoRI and BamHI sites of pMiniMAD which carries a temperature sensitive origin of replication and an erythromycin resistance cassette to generate pDP332 [98]. The plasmid pDP332 was introduced to DS2569 by single cross-over integration by transformation at the restrictive temperature for plasmid replication (37°C) using *mls* resistance as a selection. The integrated plasmid was then transduced into 3610. To evict the plasmid, the strain was incubated in 3ml LB broth at a permissive temperature for plasmid replication (22°C) for 14 hours, diluted 30-fold in fresh LB broth, and incubated at 22°C for another 8 hours. Dilution and outgrowth was repeated 2 more times. Cells were then serially diluted and plated on LB agar at 37°C. Individual colonies were patched on LB plates and LB plates containing *mls* to identify *mls* sensitive colonies that had evicted the plasmid. Chromosomal DNA from colonies that had excised the plasmid was purified and screened by PCR using primers 1692/1695 to determine which isolate had retained the *ΔfliO* allele.

To generate the *ΔfliP* in frame marker-less deletion construct, the region upstream of *fliP* was PCR amplified using the primer pair 2290/2291 and digested with EcoRI and XhoI, and the region downstream of *fliP* was PCR amplified using the primer pair 2292/2293 and digested with XhoI and BamHI. The two fragments were then simultaneously ligated into the EcoRI and BamHI sites of pMiniMAD which carries a temperature sensitive origin of replication and an erythromycin resistance cassette to generate pDP346. The plasmid pDP346 was introduced to DS2569 by single cross-over integration by transformation at the restrictive temperature for plasmid replication (37°C) using *mls* resistance as a selection. The integrated plasmid was then transduced into 3610 and evicted as described above. Chromosomal DNA from colonies that had excised the plasmid was purified and screened by PCR using primers 2292/2293 to determine which isolate had retained the *ΔfliP* allele.

To generate the *ΔcspB* in frame marker-less deletion construct, the region upstream of *cspB* was PCR amplified using the primer pair 3944/3945 and digested with EcoRI and BamH1, and the region downstream of *cspB* was PCR amplified using the primer pair 3946/3947 and digested with BamHI and XhoI. The two fragments were then simultaneously ligated into the EcoRI and SalI sites of pMiniMAD which carries a temperature sensitive origin of replication and an erythromycin resistance cassette to generate pAP94. The plasmid pAP94 was introduced to DK1042 by single cross-over integration by transformation at the restrictive temperature for plasmid replication (37°C) using *mls* resistance as a selection. The plasmid was evicted as described above and chromosomal DNA from colonies that had excised the plasmid was purified and screened by PCR using primers 3944/3947 to determine which isolate had retained the *ΔcspB* allele.

#### fliG^Q132R^ ΔfliM

To generate the *fliG* ^*sob28*^ allelic exchange construct, the region spanning the *fliG* ^*sob28*^ point mutation was PCR amplified from DS9155 using the primer pair 1229/3632 and digested with EcoRI and SalI. The fragment was then ligated into the EcoRI and SalI sites of pMiniMAD which carries a temperature sensitive origin of replication and an erythromycin resistance cassette to generate pAP80. The plasmid pAP80 was introduced to DK1042. Plasmid eviction was conducted as described above and chromosomal DNA from colonies that had excised the plasmid was purified and screened by PCR using primers 1229/3632 and sequencing to determine which isolate had retained the *fliG* ^*sob28*^ point mutation to generate DK1285. The plasmid pSG32 was then transformed into DK1285 and evicted (as described in 53) and screened by PCR using primers 1569/1572 to generate strain DK1412.

#### P_flache_^sob6^

To generate the *P*_*flache*_^*sob6*^ allelic exchange construct, the region spanning the *P*_*flache*_^*sob6*^ point mutation was PCR amplified from DS7063 using the primer pair 1648/1482 and digested with EcoRI and BamHI. The fragment was then ligated into the EcoRI and BamHI sites of pMiniMAD which carries a temperature sensitive origin of replication and an erythromycin resistance cassette to generate pAP46. The plasmid pAP46 was introduced to DS2569 by transformation and transduced to 3610. Plasmid eviction was conducted in was conducted as described above and chromosomal DNA from colonies that had excised the plasmid was purified and screened by PCR using primers 1648/1482 and sequencing to determine which isolate had retained the *P*_*flache*_^*sob6*^ point mutation.

#### P_flache_^sob21^

To generate the *P*_*flache*_^*sob21*^ allelic exchange construct, the region spanning the *P*_*flache*_^*sob21*^ point mutation was PCR amplified from DS9148 using the primer pair 1648/1482 and digested with EcoRI and BamHI. The fragment was then ligated into the EcoRI and BamHI sites of pMiniMAD which carries a temperature sensitive origin of replication and an erythromycin resistance cassette to generate pAP44. The plasmid pAP44 was introduced to DS2569 by transformation and transduced to 3610. Plasmid eviction was conducted in was conducted as described above and chromosomal DNA from colonies that had excised the plasmid was purified and screened by PCR using primers 1648/1482 and sequencing to determine which isolate had retained the *P*_*flache*_^*sob21*^ point mutation.

#### Complementation constructs

To generate the P_*flache*_-*fliO* complementation construct pAP60 and the P_*flache*_-*fliO* ^*sob22*^ complementation construct pAP61, PCR using primer pair 3217/3218 amplified *fliO* from *B*. *subtilis* 3610 and DS9149 chromosomal DNA respectively and the resulting products were digested with XhoI and BamHI. Concurrently, a PCR product containing the *flache* promoter was amplified from *B*. *subtilis* 3610 chromosomal DNA using the primer pair 2460/2461 and digested with EcoRI and XhoI. The two fragments were then simultaneously ligated into the EcoRI and BamHI sites of pAH25 containing a spectinomycin resistance cassette between two arms of the *amyE* gene (gift from Amy Camp, Mount Holyoke University).

To generate the P_*flache*_-*fliP* complementation construct pAP69 and the *P*_*flache*_ *-fliP* ^*sob22*^ complementation construct pAP71, PCR using primer pair 3449/3450 containing amplified *fliP* from *B*. *subtilis* 3610 and DS9149 chromosomal DNA respectively and the resulting products were digested with XhoI and BamHI. Concurrently, a PCR product containing the *flache* promoter was amplified from *B*. *subtilis* 3610 chromosomal DNA using the primer pair 2460/2461 and digested with EcoRI and XhoI. The two fragments were then simultaneously ligated into the EcoRI and BamHI sites of pAH25 containing a spectinomycin resistance cassette between two arms of the *amyE* gene.

Gibson isothermal assembly was used to generate the series of constructs to complement the region mutated by *sob24* as restriction enzyme based cloning strategies failed when DNA fragments containing the 5’ end of the *yhcJ* gene was introduced to *E*. *coli*. To generate arms for homologous recombination, one arm containing the *amyE* gene and upstream region was amplified with primers 3733/3180, and one arm containing the *amyE* gene, the spectinomycin resistance cassette, and the downstream region was amplified with primers 3177/4086 both using chromosomal DNA purified from DK1118 as a template. Next, the complementation fragments were amplified from 3610 template DNA as follows: “comp1” region was amplified with primers 4087/4088, “comp2” region was amplified with 4090/4088, “comp3” region was amplified using primers 4087/4089, and “comp4” region was amplified using primers 4090/4089. Each complementation region was combined with the arms from the *amyE* locus, assembled using Gibson assembly, and transformed into DK1042 selecting for spectinomycin resistance [99].

#### Inducible constructs

To generate the inducible *amyE*::*P*_*hyspank*_ *-fliP spec* construct pAP70, a PCR product containing *fliP* was amplified from *B*. *subtilis* 3610 chromosomal DNA using the primer pair 3451/3473, digested with NheI and SphI, and cloned into the NheI and SphI sites of pDR111 containing a spectinomycin resistance cassette, a polylinker downstream of the *P*_*hyspank*_ promoter, and the gene encoding the LacI repressor between the two arms of the *amyE* gene [100].

To generate the inducible *amyE*::*P*_*hyspank*_ *-fliO spec* construct pAP85, a PCR product containing *fliP* was amplified from *B*. *subtilis* 3610 chromosomal DNA using the primer pair 3741/3742, digested with NheI and SphI, and cloned into the NheI and SphI sites of pDR111.

To generate the inducible *amyE*::*P*_*hyspank*_ *-fliQ spec* construct pKRH44, a PCR product containing *fliQ* was amplified from *B*. *subtilis* 3610 chromosomal DNA using the primer pair 4560/4561, digested with NheI and SphI, and cloned into the NheI and SphI sites of pDR111.

### SPP1 phage transduction

To 0.2 ml of dense culture grown in TY broth (LB broth supplemented after autoclaving with 10 mM MgSO_4_ and 100 μM MnSO_4_), serial dilutions of SPP1 phage stock were added and statically incubated for 15 minutes at 37°C. To each mixture, 3 ml TYSA (molten TY supplemented with 0.5% agar) was added, poured atop fresh TY plates, and incubated at 30°C overnight. Top agar from the plate containing near confluent plaques was harvested by scraping into a 15 ml conical tube, vortexed, and centrifuged at 5,000 x g for 5 minutes. The supernatant was treated with 25 μg/ml DNase before being passed through a 0.45 μm syringe filter and stored at 4°C.

Recipient cells were grown to stationary phase in 3 ml TY broth at 37°C. 1 ml cells were mixed with 25 μl of SPP1 donor phage stock. 9 ml of TY broth was added to the mixture and allowed to stand at 37°C for 30 minutes. The transduction mixture was then centrifuged at 5,000 x g for 5 minutes, the supernatant was discarded and the pellet was resuspended in the remaining volume. 100 μl of cell suspension was then plated on LB fortified with 1.5% agar, the appropriate antibiotic, and 10 mM sodium citrate.

### Swarm expansion assay

Cells were grown to mid-log phase at 37°C in LB broth and resuspended to 10 OD_600_ in pH 8.0 PBS buffer (137 mM NaCl, 2.7 mM KCl, 10 mM Na_2_HPO_4_, and 2 mM KH_2_PO_4_) containing 0.5% India ink (Higgins). Freshly prepared LB containing 0.7% Bacto agar (25 ml/plate) was dried for 10 minutes in a laminar flow hood, centrally inoculated with 10 μl of the cell suspension, dried for another 10 minutes, and incubated at 37°C [101]. The India ink demarks the origin of the colony and the swarm radius was measured relative to the origin. For consistency, an axis was drawn on the back of the plate and swarm radii measurements were taken along this transect. For experiments including IPTG, cells were propagated in broth in the presence of IPTG, and IPTG was included in the swarm agar plates.

#### Western blotting

*B*. *subtilis* strains were grown in LB broth to OD_600_ ~0.5, 1 ml was harvested by centrifugation, and resuspended to 10 OD_600_ in Lysis buffer (20 mM Tris pH 7.0, 10 mM EDTA, 1 mg/ml lysozyme, 10 g/ml DNAse I, 100 g/ml RNAse I, 1 mM PMSF) and incubated 30 minutes at 37°C. Each lysate was then mixed with the appropriate amount of 6x SDS loading dye to dilute the loading dye to 1x concentration. Samples were separated by 12% Sodium dodecyl sulfate-polyacrylamide gel electrophoresis (SDS-PAGE). The proteins were electroblotted onto nitrocellulose and developed with a 1:20,000 dilution of (anti-FliG, anti-FlgE), 1:10,000 dilution of (anti-SigD), or 1:80,000 dilution of (anti-Hag, anti-SigA) of primary antibody and a 1:10,000 dilution secondary antibody (horseradish peroxidase-conjugated goat anti-rabbit immunoglobulin G). Immunoblot was developed using the Immun-Star HRP developer kit (Bio-Rad).

### FlgE secretion assay

For the pellet fraction (cytoplasmic and cell-associated proteins), *B*. *subtilis* strains were grown in 20 ml LB broth in the presence 1 mM IPTG when appropriate to an OD_600_ of 1.2–1.7, and 10 ml samples of broth culture were harvested by centrifugation, resuspended to 10 OD_600_ units in lysis buffer (20 mM Tris [pH 7.0], 10 mM EDTA, 1 mg/ml lysozyme, 10 μg/ml RNase I, 1 mM PMSF), and incubated 30 minutes at 37°C. For the supernatant fraction (secreted extracellular proteins), 10 ml of supernatant was collected from the same cultures as those used to generate the pellet fractions. The supernatant was clarified by centrifugation at 5,000 × *g* for 30 min and treated with 1 ml of freshly prepared 0.015% sodium deoxycholate for 10 min at room temperature. Proteins from the supernatant were precipitated by adding 500 μl chilled trichloroacetic acid (TCA) and incubating the mixture for >2 h on ice at 4°C. Precipitated proteins were pelleted at 9,447 × *g* for 10 min at 4°C, washed twice with 1 ml ice-cold acetone, and resuspended to 10 OD_600_ units in 0.1 N sodium hydroxide. Ten microliters of cell pellet or supernatant sample was mixed with 2 μl 6× SDS loading buffer. Samples were separated in parallel by 15% SDS-PAGE. Proteins were electroblotted onto nitrocellulose for 1 hour at 400 mA and probed with a 1:20,000 dilution of anti-FlgE primary antibody and with a 1:10,000 dilution of secondary antibody (horseradish peroxidase [HRP]-conjugated goat anti-rabbit immunoglobulin G). Immunoblots were developed using the Pierce ECL Western blotting substrate kit (Thermo Scientific).

### Microscopy

Fluorescence microscopy was performed with a Nikon 80i microscope along with a phase contrast objective Nikon Plan Apo 100X and an Excite 120 metal halide lamp. FM4-64 was visualized with a C-FL HYQ Texas Red Filter Cube (excitation filter 532–587 nm, barrier filter >590 nm). GFP was visualized using a C-FL HYQ FITC Filter Cube (FITC, excitation filter 460–500 nm, barrier filter 515–550 nm). Images were captured with a Photometrics Coolsnap HQ^2^ camera in black and white, false colored and superimposed using Metamorph image software.

For *P*_*hag*_ *-GFP* microscopy, cells were grown at 37°C in LB broth to OD_600_ 0.6–1.0, resuspended in 30 μl PBS buffer containing 5 μg/ml FM 4–64 and incubated for 5 min at room temperature. The cells were pelleted, resuspeneded in 30μl PBS buffer, and were observed by spotting 4 μl of suspension on a cleaned microscope slide and immobilized with a poly-L-lysine-treated glass coverslip.

For fluorescent microscopy of flagellar filaments, 1.0 ml of broth culture was harvested at 0.6–1.0 OD_600_, resuspended in 50 μl of PBS buffer containing 5μg/ml Alexa Fluor 488 C_5_ maleimide (Molecular Probes), incubated for 3 min at room temperature, and washed once in 1.0 ml of PBS buffer. The suspension was pelleted, resuspended in 30 μl of PBS buffer containing 5 μg/ml FM 4–64, and incubated for 5 min at room temperature. The cells were pelleted, resuspeneded in 30 μl PBS buffer, and were observed by spotting 4 μl of suspension on a cleaned microscope slide and immobilized with a poly-L-lysine-treated glass coverslip.

For fluorescent microscopy of flagellar hooks, 1.0 ml of broth culture was harvested at 0.6–1.0 OD_600_, resuspended in 50 μl of PBS buffer containing 5μg/ml Alexa Fluor 488 C_5_ maleimide (Molecular Probes), incubated for 3 min at room temperature, and washed once in 1.0 ml of PBS buffer. The suspension was pelleted, resuspended in 30 μl of PBS buffer containing 5 μg/ml FM 4–64, and incubated for 5 min at room temperature. The cells were pelleted, resuspeneded in 30 μl PBS buffer, and were observed by spotting 4 μl of suspension on a cleaned microscope slide and immobilized with a poly-L-lysine-treated glass coverslip.

For P_*fliM*_-*fliM-GFP* microscopy, cells were grown at 37°C in LB broth to OD_600_ 0.6–1.0, resuspended in 30 μl PBS buffer containing 5 μg/ml FM 4–64 and incubated for 5 min at room temperature. The cells were pelleted, resuspeneded in 30μl PBS buffer, and were observed by spotting 4 μl of suspension on a cleaned microscope slide and immobilized with a poly-L-lysine-treated glass coverslip.

For super-resolution microscopy, the OMX 3D-SIM Super-Resolution system was used. Supper-resolution microscopy was performed by using a 1.4-numerical-aperture (NA) Olympus 100X oil objective. FM4-64 was observed using laser line 561 and emission filter 609 nm to 654 nm, and GFP (along with Alexa Fluor 488 nm) was observed using laser line 488 nm and emission filter 500 nm to 550 nm. Images were captured using a Photometrics Cascade II electron-multiplying charge-coupled-device camera, processed using SoftWorx imaging software, and analyzed using Imaris software.

### OMX 3D-SIM counts

Images that were processed as described above were used in Imaris (Bitplane) to determine the number and location of FliM-GFP or FlgE labeled with a fluorescent dye (FlgE^T123C^). The spots feature within the software labelled each puncta by the search parameter of identifying spots of 1 μM in the 488 nm wavelength. Cell pole positions were determined by using the 561 nm wavelength using the slice feature. The x, y, and z coordinates of each puncta in each cell was exported from Imars. Scripts were developed in MATLAB (The Mathworks) for importing two-dimensional Cartesian coordinates for basal body, hook, and cell lengths from Imaris.

### β-Galactosidase assays

Cells were harvested from cultures growing at 37°C in LB broth. Cells were collected in 1.0 ml aliquots and suspended in an equal volume of Z buffer (40 mM NaH_2_PO_4_, 60 mM NaHPO_4_, 1.0 mM MgSO_4_, 10 mM KCl, and 38 mM 2-mercaptoethanol). Lysozyme was added to each sample to a final concentration of 0.2 mg/ml and incubated at 37°C for 30 min. Each sample was diluted in Z buffer to a final volume of 500 μl, and the reaction was started with 100 μl of 4 mg/ml 2-nitrophenyl β-galactopyranoside in Z buffer and stopped with 250 μl of 1M Na_2_CO_3_. The OD_420_ of the reaction mixture was measured, and the β-galactosidase-specific activity was calculated according the equation [OD_420_/(time X OD_600_)] X dilution factor X 1000.

### Direct Sanger sequencing

For *sob* and *soa* mutations contained within the *flache* promoter, a PCR product containing the *flache* promoter was amplified from *B*. *subtilis* chromosomal DNA (either from strain 3610 or the appropriate suppressor strain) using the primer set 1921/1922. The *P*_*flache*_ PCR product was then sequenced using primer 1921 and 1922 individually.

### Library construction and genome sequencing

Genomic samples were fragmented using the Corvaris S220 ultrasonicator and then assayed using the Agilent TapeStatin using D1K HS tapes. The fagmenented samples were processed into Illumina DNA-Seq libraries using Bechman SWHT chemistry in conjunction with BioScientific NextFlex Adaptors on the BIomekFx automated workstation. The large (350bp-750bp) size selection option was selected, and 12 μl of preamplified library was used as template in a 10 cycle amplification reaction. Following amplification, the libraries were cleaned using a 1X AmpureXP ratio and eluted in EB buffer.

After Illumina sequencing, MiSeq reads were trimmed using a quality cutoff of 20 and remaining sequencing adapters were removed using Cutadapt 1.2.1. FLASH 1.2.2 was then used to merge read pairs in which the forward and reverse read overlapped. The assemblies were performed using Newbler 2.7 using the merged reads as singletons and the unmerged reads as paired end reads. For SNP prediction, reads were mapped against both the NCIB 3610 reference and the DS234 assembly. Mapping was performed with bowtie 2.0.2 using the default parameters. Samtools was used to convert the mapping data to a pileup format. VarScan 2.3.2 was used to call SNPs, using parameters to require a minimum read depth of 20 and a minimum variant frequency of 90%.

To verify the point mutation in *sob28*, A PCR product containing *fliG* was amplified from *B*. *subtilis* chromosomal DNA strain DS9155 using the primer set 1229/3632. The *fliG* PCR product was then sequenced using primer 788 to verify the *sob* mutation. To verify the deletion boundaries within *sob24* and *sob25*, A PCR product containing the deletion region was amplified from *B*. *subtilis* chromosomal DNA strain DS9151 and DS9152 using the primer set 3628/3629. The PCR product was then sequenced using primer 3476 to verify the deletion boundaries.

### SPP1-mediated phage co-transduction linkage mapping

To determine the frequency of co-transduction of the *sob22* mutation to the *swrB*::*tet* allele, a lysate was generated on the suppressor strain (DS9149) and the *swrB*::*tet* allele was transduced to the wildtype 3610 strain. Three hundred of the resulting colonies were then picked onto 0.7% LB swarm agar plates to enumerate the number of swarm proficient colonies. The percentage of colonies that were swarm proficient was inversely proportional to the distance between the *swrB*::*tet* and the suppressor mutation. The primer set 1692/2293 was used to generate a PCR product 13kb upstream of the *swrB*::*tet* allele within the *flache* operon. The *flache* PCR product was then sequenced using primer 1601.

### RNA purification

25 ml of cells were grown in triplicate in LB broth at 37°C until the cultures reached 1.0 OD_600_. 10 ml of each culture was then harvested into 15 ml conical tubes containing 1.25 ml of stop solution (-20°C—5% phenol diluted in 100% ethanol) and centrifuged at 5,800 rpm for 10 min at 4°C. The resulting pellets were then transferred to 1.5 ml microfuge tubes containing 500 μl of -80°C methanol, centrifuged at 14,000 rpm for 1 min at 4°C, and then stored at -80°C. The pellets where then resuspended in 850 μl TE buffer and then transferred to microfuge tubes containing 10 mg/ml lysozyme, inverted 4–6 times and placed at 37°C for 45 min. After this incubation, 50 μl of 10% SDS (Sodium dodecyl sulfate) was as added to the samples, the samples were inverted 4–6 times, and then 50 μl of 3M sodium acetate, pH 5.2 was added and the tubes were again inverted 4–6 times. Each sample was then split into to 500 μl volumes into separate microfuge tubes. 500 μl of phenol was added to each sample, the samples were inverted 10 times, and then placed in a 64°C water bath for 6 min. The tubes were inverted every minute during this water bath incubation. After the water bath incubation, the samples were centrifuged at 14,000 rpm for 10 min at 4°C. The resulting aqueous layer was then transferred to a fresh 1.5 ml microfuge tube where an equal volume of chloroform was added. The tubes were inverted 6–10 times, and then centrifuged at 14,000 rpm for 5 min at 4°C. The resulting aqueous layer was then transferred to a fresh 1.5 ml microfuge tubes where 1/10 the volume of 3M sodium acetate, pH 5.2 and 2 volumes of -20°C 100% ethanol were added to the tubes. The resulting mixtures were incubated at -80°C for 30 min and then centrifuged at 14,000 rpm for 40 min at 4°C. The ethanol layer was then removed, and the resulting white pellet was washed with 1 ml of -20°C 75% ethanol, centrifuged at 14,000 rpm for 5 min at 4°C. The ethanol layer was removed and the pellet was resuspended in 50 μl of RNase free water, and each split pool was rejoined together an incubated at 50°C to encourage resuspension.

### Quantitative PCR primer optimization

Each primer pair was diluted to both 1 μM and 5 μM stocks and subsequently mixed into 25 separate reactions in duplicate. Each of the 25 reactions was a permutation of one forward primer at either 50 nM, 100 nM, 300 nM, 600 nM, or 900 nM concentration with its corresponding reverse primer at either 50 nM, 100 nM, 300 nM, 600 nM, or 900 nM concentration along with SYBR Green SuperMix reagent (Quanta Biosciences), and 10^6^ copies of template. Each primer optimization assay also contained two additional control reactions containing forward and reverse primers each at either 50nM or 900 nM, Green SuperMix reagent (Quanta Biosciences) and no template. Quantitative PCR was conducted on each reaction set for each primer used in downstream quantitative PCR assays on the Stratagene MX3500 Pro thermocycler. Data were analyzed using the MXPro Stratagene software package.

### Quantitiative reverse transcriptase PCR

Total RNA was isolated as described above. Isolated RNA was DNase digested used TURBO DNA free ket (Ambion). cDNA was reverse transcribed from each DNase-digested RNA sample using random dT primers of the qScript cDNA superMix (Quanta Biosciences). Quantitave PCR was performed with specific primer pairs whose concentrations were optimized as described above and either diluted cDNA templeate, DNase digested RNA, or no template using SYBR Green SuperMix (Quanta Biosciences) on the Stratagene MX3500 Pro thermocycler. Data were analyzed using the MXPro Stratagene software package. The following primers where used: 1448/1449 (*sigD*), 1450/1451 (*sigA)*, 1560/1561 (*flgB*), 1562/1563 (*cheD*), 1564/1565 (*hag*), 1596/1597 (*fliF*), 1598/1599 (*flgE*), 1602/1603 (*flhA*), 1604/1605 (*cheB*), 1652/1653 (*fliI*), 1654/1655 (*fliK*), 1656/1657 (*fliY*), 1658/1659 (*fliR*), 1662/1663 (*cheW*), and 3758/3759 (*fliP*)

## Supporting Information

S1 Fig Cells mutated for SwrA and SwrB have a reduced frequency of *P*_*hag*_ *-GFP* expression. Sample of raw fluorescence microscopy images to support Fig 2C. Membranes stained with FM4-64 and false colored red. Fluorescence from the *P*_*hag*_ *-GFP* reporter false colored green. Images were taken at 40X and scale bar is 20 μm.
(EPS)

S2 Fig Suppressors of *swrB* (*sob*) and *swrA* (*soa*) in the *P*_*fla/che*_ promoter region. A) A map of the region surrounding the *P*_*fla/che*_ promoter. Large open arrows represent open reading frames. Bent arrows indicate promoters. Lollipop indicates the rho-independent terminator downstream of the *codY* gene. Red and blue bars indicate the boundaries of deletion in *sob* and *soa* mutants respectively. B) Sequence surrounding the *P*_*fla/che*_ promoter. The -10 and -35 elements of the σ^A^ dependent *P*_*fla/che*_ promoter and the σ^D^-dependent *P*_*D3*_ promoter are indicated. The +1 indicates the transcriptional start site for the *fla/che* operon. The location of the DegU binding site is indicated [57]. The location and nature of the *sob* (red) and *soa* (blue) mutations are indicated below the sequence.
(EPS)

S3 Fig The *sob6* allele does not mutate a *trans* factor. Quantitative swarm expansion assays of the indicated genetic backgrounds. Genotypes in parentheses indicate complementation constructs. The following strains were used the generate panels A and B: *swrB sob6* (DS7063), *swrB sob6* (*P*_*flache*_^*WT*^) (DS9974), *swrB* (DS1107), and *swrB* (*P*_*flache*_^*sob6*^) (DK21). Each point is the average of three replicates.
(EPS)

S4 Fig The *sob24* allele does not increase expression from the Pfla/che promoter. β-galactosidase assays of *lacZ* gene expression under the control of WT *P*_*fla/che*_ promoter regions. Each reporter was expressed in either WT (DS793), *swrB* (DS1461), or *swrB sob24* (DK1966) mutant backgrounds as indicated. Error bars are the standard deviation of three replicates.
(EPS)

S5 Fig Overexpression of *fliQ* does not restore swarming to a *swrB* mutant. Swarm expansion assays of a *fliQ* mutant (left, DK3350) and a *swrB* mutant (right, DK3354) containing an ectopic IPTG-inducible copy of the fliQ in the presence (closed symbols) and absence (open symbols) of 1 mM IPTG. Points are the average of three replicates.
(EPS)

S6 Fig Cells defective for the C-ring do not synthesize flagellar filaments and filament synthesis cannot be rescued by the *fliG* ^*Q132R*^ allele. Fluorescence micrographs of cells stained for membranes with FM4-64 (false colored red) and for the Hag^T209C^ allele with fluorescent maleimide (false colored green). The following strains were used to generate this panel: *fliM* (DK838), *fliM fliG* ^*Q132R*^ (DK839), and *fliG* ^*Q132R*^ (DK1978).
(EPS)

S1 Table Plasmids. (DOCX)

S2 Table Primers. (DOCX)

S3 Table β-galactosidase activities to support Fig 2B ^a^. (DOCX)

S4 Table β-galactosidase activities to support Fig 6 ^a^. (DOCX)

S5 Table Supplemental strains. (DOCX)

## References

[1] Macnab RM (2003) How bacteria assemble flagella. Annu Rev Microbiol 57:77–100. 12730325 10.1146/annurev.micro.57.030502.090832

[2] Chevance FFV, Hughes KT (2008) Coordinating assembly of a bacterial macromolecular machine. Nat Rev Microbiol 6:455–465. 10.1038/nrmicro1887 18483484 PMC5963726

[3] Mukherjee S, Kearns DB (2015) The structure and regulation of flagella in *Bacillus subtilis* . Annu. Rev. Genet. 48:319–340. 10.1146/annurev-genet-120213-092406 PMC4869327 25251856

[4] Ueno T, Oosawa K, Aizawa S-I (1992) M ring, S ring and proximal rod of the flagellar basal body of *Salmonella typhimurium* are composed of subunits of a single protein, FliF. J Mol Biol 227:672–677. 1404383 10.1016/0022-2836(92)90216-7

[5] Fan F, Ohnishi K, Francis NR, Macnab RM. (1997) The FliP and FliR proteins of *Salmonella typhimurium*, putative components of the type III flagellar export apparatus, are located in the flagellar basal body. Mol. Microbiol. 26:1035–1046. 9426140 10.1046/j.1365-2958.1997.6412010.x

[6] Minamino T, Macnab RM (1999) Components of the *Salmonella* flagellar export apparatus and classification of export substrates. J Bacteriol 181:1388–1394. 10049367 10.1128/jb.181.5.1388-1394.1999 PMC93525

[7] Li H, Sourjik V (2011) Assembly and stability of flagellar motor in *Escherichia coli* . Mol Microbiol 80:886–899. 10.1111/j.1365-2958.2011.07557.x 21244534

[8] Blair DF (2003) Flagellar movement driven by proton translocation. FEBS Lett 545:86–95. 12788496 10.1016/s0014-5793(03)00397-1

[9] Paul K, Brunstetter D, Titen S, Blair DF (2011) A molecular mechanism of direction switching in the flagellar motor of *Escherichia coli* . Proc Natl Acad Sci USA 108:17171–17176. 10.1073/pnas.1110111108 21969567 PMC3193218

[10] Abby SS, Rocha EPC (2012) The non-flagellar type III secretion system evolved from the bacterial flagellum and diversified into host-cell adapted systems. PLoS Genet 8:e1002983 10.1371/journal.pgen.1002983 23028376 PMC3459982

[11] Erhardt M, Namba K, Hughes KT (2010) Bacterial nanomachines: the flagellum and type III injectisome. Cold Spring Harb Perspect Biol 2:a000299 10.1101/cshperspect.a000299 20926516 PMC2964186

[12] Wagner S, Königsmaier L, Lara-Tejero M, Lefebre M, Marlovits TC, Galán JE (2010) Organization and coordinated assembly of the type III secretion export apparatus. Proc Natl Acad Sci USA 107:17745–17750. 10.1073/pnas.1008053107 20876096 PMC2955140

[13] Diepold A, Amstutz M, Abel S, Sorg I, Jenal U, Cornelis GR (2010) Deciphering the assembly of the *Yersinia* type III secretion injectisome. EMBO J 29:1928–1940. 10.1038/emboj.2010.84 20453832 PMC2885934

[14] Pallen MJ, Penn CW, Chaudhuri RR (2005) Bacterial flagellar diversity in the post-genomic era. Trends Microbiol 13:143–149. 15817382 10.1016/j.tim.2005.02.008

[15] Minamino T, Macnab RM (2000) Domain structure of *Salmonella* FlhB, a flagellar export component responsible for substrate specificity switching. J Bacteriol 182:4906–4914. 10940035 10.1128/jb.182.17.4906-4914.2000 PMC111371

[16] Fraser GM, Hirano T, Ferris HU, Devgan LL, Kihara M, Macnab RM (2003) Substrate specificity of type III flagellar protein is *Salmonella* is controlled by subdomain interactions in FlhB. Mol Microbiol 48:1043–1057. 12753195 10.1046/j.1365-2958.2003.03487.x

[17] Bange G, Kümmerer N, Engel C, Bozkurt G, Wild K, Sinning I (2010) FlhA provides the adaptor for coordinated delivery of late flagella building blocks to the type III secretion system. Proc Natl Acad Sci USA 107:11295–11300. 10.1073/pnas.1001383107 20534509 PMC2895114

[18] Kinoshita M, Hara N, Imada K, Namba K, Minamino T (2013) Interactions of bacterial flagellar chaperone-substrate complexes with FlhA contribute to co-ordinating assembly of the flagellar filament. Mol Microbiol 90:1249–1261. 10.1111/mmi.12430 24325251

[19] Minamino T, Namba K (2008) Distinct roles of the FliI ATPase and proton motive force in bacterial flagellar protein export. Nature 451:485–488. 10.1038/nature06449 18216858

[20] Paul K, Erhardt M, Hirano T, Blair DF, Hughes KT (2008) Energy source of flagellar type III secretion. Nature 451:489–492. 10.1038/nature06497 18216859

[21] Barker CS, Meshcheryakova IV, Kostyukova AS, Samatey FA (2010) FliO regulation of FliP in the formation of the *Salmonella enterica* flagellum. PLoS Genet 6:e1001143 10.1371/journal.pgen.1001143 20941389 PMC2947984

[22] Barker CS, Meshcheryakova IV, Inoue T, Samatey FA. (2014) Assembling flagella in *Salmonella* mutant strains producing a type III export apparatus without FliO. J. Bacteriol. 196:4001–4011. 10.1128/JB.02184-14 25201947 PMC4248865

[23] Iyoda S, Kutsukake K (1995) Molecular dissection of the flagellum-specific anti-sigma factor, FlgM, of *Salmonella typhimurium* . Mol Gene Genet 249:417–424. 10.1007/BF00287103 8552046

[24] Chilcott GS, Hughes KT (1998) The type III secretion determinants of the flagellar anti-transcription factor, FlgM, extend from the amino terminus in the anti-σ^28^ domain. Mol Microbiol 30:1029–1040. 9988479 10.1046/j.1365-2958.1998.01131.x

[25] Hirano T, Minamino T, Namba K, Macnab RM (2003) Substrate specificity classes and the recognition signal for *Salmonella* type III flagellar export. J Bacteriol 185:2485–2492. 12670972 10.1128/JB.185.8.2485-2492.2003 PMC152621

[26] Auvray F, Thomas J, Fraser GM, Hughes C (2001) Flagellin polymerization control by a cytoplasmic export chaperone. J Mol Biol 308:221–229. 11327763 10.1006/jmbi.2001.4597 PMC2528291

[27] Ferris HU, Minamino T (2006) Flipping the switch: bringing order to flagellar assembly. Trends Microbiol 14:519–526. 17067800 10.1016/j.tim.2006.10.006

[28] Minamino T, Kinoshita M, Hara N, Takeuchi S, Hida A, Koya S, Glenwright H, Imada K, Aldridge PD, Namba K (2012) Interaction of a bacterial flagellar chaperone FlgN with FlhA is required for efficient export of its cognate substrates. Mol Microbiol 83:775–788. 10.1111/j.1365-2958.2011.07964.x 22233518

[29] Hughes KT, Gillen KL, Semon MJ, Karlinsey JE (1993) Sensing structural intermediates in bacterial flagellar assembly by export of a negative regulator. Science 262:1277–1280. 8235660 10.1126/science.8235660

[30] Kutsukake K (1994) Excretion of the anti-sigma factor through a flagellar substructure couples flagellar gene expression with flagellar assembly in *Salmonella typhimurium* . Mol Gen Genet 243:605–612. 8028576 10.1007/BF00279569

[31] Sockett H, Yamaguchi S, Kihara M, Irikura VM, Macnab RM (1992) Molecular analysis of the flagellar switch protein FliM of *Salmonella typhimurium* . J Bacteriol 174:793–806. 1732214 10.1128/jb.174.3.793-806.1992 PMC206156

[32] Irikura VM, Kihara M, Yamaguchi S, Sockett H, Macnab RM (1993) *Salmonella typhimurium fliG* and *fliN* mutations causing defects in assembly, rotation, and switching of the flagellar motor. J Bacteriol 175:802–810. 8423152 10.1128/jb.175.3.802-810.1993 PMC196220

[33] Lloyd SA, Tang H, Wang X, Billing S, Blair DF (1996) Torque generation in the flagellar motor of *Escherichia coli*: evidence of a direct role for FliG but not FliM or FliN. J Bacteriol 178:223–331. 8550421 10.1128/jb.178.1.223-231.1996 PMC177643

[34] González-Pedrajo B, Minamino T, Kihara M, Namba K (2006) Interactions between C-ring proteins and export apparatus components: a possible mechanism for facilitating type III protein export. Mol. Microbiol. 60:984–998. 16677309 10.1111/j.1365-2958.2006.05149.x

[35] McMurray JL, Murphy JW, González-Pedrajo B. (2006) The FliN-FliH interaction mediates localization of flagellar export ATPase FliI to the C ring complex. Biochemistry 45:11790–11798. 17002279 10.1021/bi0605890

[36] Paul K, Harmon JG, Blair DF. (2006) Mutational analysis of the flagellar rotor protein FliN: identification of surfaces important for flagellar assembly and switching. J. Bacteriol. 188:5240–5248. 16816196 10.1128/JB.00110-06 PMC1539977

[37] Minamino T, Yoshimura SD, Morimoto YV, González-Pedrajo B, Kami-Ike N, Namba K. (2009) Roles of the extreme N-terminal region of FliH for efficient localization of the FliH-FliI complex to the bacterial flagellar type III export apparatus. Mol. Microbiol. 74:1471–1483. 10.1111/j.1365-2958.2009.06946.x 19889085

[38] Kearns DB, Chu F, Rudner R, Losick R (2004) Genes governing swarming in *Bacillus subtilis* and evidence for a phase variation mechanism controlling surface motility. Mol Microbiol 52:357–369. 15066026 10.1111/j.1365-2958.2004.03996.x

[39] Werhane H, Lopez P, Mendel M, Zimmer M, Ordal GW, Márquez-Magaña LM (2004) The last gene of the *fla/che* operon in *Bacillus subtilis*, *ylxL*, is required for maximal σ^D^ function. J Bacteriol 186:4025–4029. 15175317 10.1128/JB.186.12.4025-4029.2004 PMC419943

[40] Calvio C, Celandroni F, Ghelardi E, Amati G, Salvetti S, Ceciliani F, Galizzi A, Senesi S (2005) Swarming differentiation and swimming motility in *Bacillus subtilis* are controlled by *swrA*, a newly identified dicistronic operon. J Bacteriol 187:5356–5366. 16030230 10.1128/JB.187.15.5356-5366.2005 PMC1196031

[41] Kearns DB, Losick R (2005) Cell population heterogeneity during growth of *Bacillus subtilis* . Genes Dev 19:3083–3094. 16357223 10.1101/gad.1373905 PMC1315410

[42] Mukherjee S, Bree AC, Liu J, Patrick JE, Chien P, and Kearns DB. (2015) Adaptor-mediated Lon proteolysis restricts *Bacillus subtilis* hyperflagellation. Proc. Natl. Acad. Sci. USA. 112:250–255. 10.1073/pnas.1417419112 25538299 PMC4291670

[43] Blair KM, Turner L, Winkelman JT, Berg HC, Kearns DB (2008) A molecular clutch disables flagella in the *Bacillus subtilis* biofilm. Science 320:1636–1638. 10.1126/science.1157877 18566286

[44] Mirel DB, Chamberlin MJ (1989) The *Bacillus subtilis* flagellin gene (*hag*) is transcribed by the σ^28^ form of RNA polymerase. J Bacteriol 171:3095–3101. 2498284 10.1128/jb.171.6.3095-3101.1989 PMC210020

[45] Ohnishi K, Kutsukake K, Suzuki H, Iino T (1992) A novel transcriptional regulation mechanism in the flagellar regulon of *Salmonella typhimurium*: an anti-sigma factor inhibits the activity of the flagellum-specific sigma factor, σ^F^ . Mol Microbiol 6:3149–3157. 1453955 10.1111/j.1365-2958.1992.tb01771.x

[46] Caramori T, Barillà D, Nessi C, Sacchi L, Galizzi A (1996) Role of FlgM in σ^D^-dependent gene expression in *Bacillus subtilis* . J Bacteriol 178:311–3118. 10.1128/jb.178.11.3113-3118.1996 PMC178060 8655488

[47] Fredrick K, Helmann JD (1996) FlgM is a primary regulator of σ^D^ activity, and its absence restores motility to a *sinR* mutant. J Bacteriol 178:7010–7013. 8955328 10.1128/jb.178.23.7010-7013.1996 PMC178607

[48] Bertero MG, Gonzales B, Tarricone C, Ceciliani F, Galizzi A (1999) Overproduction and characterization of the *Bacillus subtilis* anti-sigma factor FlgM. J Biol Chem 274:12103–12107. 10207036 10.1074/jbc.274.17.12103

[49] Cozy LM, Kearns DB (2010) Gene position in a long operon governs motility development in *Bacillus subtilis* . Mol Microbiol 76:273–285. 10.1111/j.1365-2958.2010.07112.x 20233303 PMC2911795

[50] Cozy LM, Phillips AM, Calvo RA, Bate AR, Hsueh Y-H, Bonneau R, Eichenberger P, Kearns DB (2012) SlrA/SinR/SlrR inhibits motility gene expression upstream of a hypersensitive and hysteretic switch at the level of σ^D^ in *Bacillus subtilis* . Mol Microbiol 83:1210–1228. 10.1111/j.1365-2958.2012.08003.x 22329926 PMC3303961

[51] Courtney CR, Cozy LM, Kearns DB (2012) Molecular characterization of the flagellar hook in *Bacillus subtilis* . J Bacteriol 194:4619–4629. 10.1128/JB.00444-12 22730131 PMC3415477

[52] Calvo R, and Kearns DB. (2015) FlgM is secreted by the flagellar export apparatus in *Bacillus subtilis* . J. Bacteriol. 197:81–91. 10.1128/JB.02324-14 25313396 PMC4288692

[53] Guttenplan SB, Shaw S, Kearns DB (2013) The cell biology of peritrichous flagella in *Bacillus subtilis* . Mol Microbiol 87:211–229. 10.1111/mmi.12103 23190039 PMC3538361

[54] Amati G, Bisicchia P, Galizzi A (2004) DegU-P represses expression of the motility *fla-che* operon in *Bacillus subtilis* . J Bacteriol 186:6003–6014. 15342569 10.1128/JB.186.18.6003-6014.2004 PMC515139

[55] Márquez-Magaña LM, Chamberlin MJ (1994) Characterization of the *sigD* transcriptional unit of *Bacillus subtilis* . J Bacteriol 176:2427–2434. 8157612 10.1128/jb.176.8.2427-2434.1994 PMC205368

[56] West JT, Estacio W, Marquez-Magana L (2000) Relative roles of the *fla/che* P_A_, P_D-3_, and *P* _*sigD*_ promoters in regulating motility and sigD expression in *Bacillus subtilis* . J Bacteriol 182:4841–4848. 10940026 10.1128/jb.182.17.4841-4848.2000 PMC111362

[57] Tsukahara K, Ogura M. (2008) Promoter selectivity of the *Bacillus subtilis* response regulator DegU, a positive regulator of the *fla/che* operon and *sacB* . BMC Microbiol. 8:8 10.1186/1471-2180-8-8 18197985 PMC2245950

[58] Ogura M, Tsukahara K (2012) SwrA regulates assembly of *Bacillus subtilis* DegU via its interaction with N-terminal domain of DegU. J Biochem 6:643–655. 10.1093/jb/mvs036 22496484

[59] Mordini S, Osera C, Marini S, Scavone F, Bellazzi R, Galizzi A, Calvio C (2013) The role of SwrA, DegU and P_D3_ in *fla/che* expression in *B*. *subtilis* . PLoS One 8:e85065/ 10.1371/journal.pone.0085065 24386445 PMC3874003

[60] Willimsky G, Bang H, Fischer G, Marahiel MA (1992) Characterization of *cspB*, a *Bacillus subtilis* inducible cold shock gene affecting cell viability at low temperatures. J Bacteriol 174:6326–6335. 1400185 10.1128/jb.174.20.6326-6335.1992 PMC207576

[61] Graumann P, Marahiel MA (1994) The major cold shock protein of *Bacillus subtilis* CspB binds with high affinity to the ATTGG- and CCAAT sequences in single stranded oligonucleotides. FEBS Lett 338:157–160. 8307174 10.1016/0014-5793(94)80355-2

[62] Vellanoweth RL, Rabinowitz JC (1992) The influence of ribosome-binding-site elements on translational efficiency in *Bacillus subtilis* and *Escherichia coli in vivo* . Mol Microbiol 6:1105–1114. 1375309 10.1111/j.1365-2958.1992.tb01548.x

[63] Brown PN, Terrazas M, Paul K, Blair DF (2007) Mutational analysis of the flagellar protein FliG: sites of interaction with FliM and implications for organization of the switch complex. J Bacteriol 189:305–312. 17085573 10.1128/JB.01281-06 PMC1797384

[64] Lee LK, Ginsburg MA, Crovace C, Donohoe M, Stock D (2010) Structure of the torque ring of the flagellar motor and the molecular basis for rotational switching. Nature 996–1000. 10.1038/nature09300 PMC3159035 20676082

[65] Ohnishi K, Ohto Y, Aizawa S, Macnab RM, Iino T. (1994) FlgD is a scaffolding protein needed for flagellar hook assembly in *Salmonella typhimurium* . J Bacteriol 176:2272–2281. 8157595 10.1128/jb.176.8.2272-2281.1994 PMC205349

[66] Jones CJ, and Macnab RM. (1990) Flagellar assembly in *Salmonella typhimurium*: analysis with temperature-senstive mutants. J. Bacteriol. 172:1327–1339. 2407720 10.1128/jb.172.3.1327-1339.1990 PMC208602

[67] Morimoto YV, Ito M, Kiraoka KD, Che Y-S, Bai F, Kami-ike N, Namba K, Minamino T. (2014) Assembly and stoichiometry of FliF and FlhA in Salmonella flagellar basal body. Mol. Microbiol. 91:1214–1226. 10.1111/mmi.12529 24450479

[68] Ohnishi K, Fan F, Schoenhals GJ, Kihara M, Macnab RM. (1997) The FliO, FliP, FliQ, and FliR proteins of *Salmonella*: putative components for flagellar assembly. J. Bacteriol. 179:6092–6099. 9324257 10.1128/jb.179.19.6092-6099.1997 PMC179513

[69] Bischoff DS, Weinreich MD, Ordal GW (1992) Nucleotide sequences of Bacillus subtilis flagellar biosynthetic genes *fliP* and *fliQ* and identification of a novel flagellar gene, *fliZ* . J Bacteriol 174:4017–4025. 1597417 10.1128/jb.174.12.4017-4025.1992 PMC206111

[70] Francis NR, Irikura VM, Yamaguchi S, DeRosier DJ, Macnab RM (1992) Localization of the *Salmonella typhimurium* flagellar switch protein FliG to the cytoplasmic M-ring face of the basal body. Proc Natl Acad Sci USA 89:6304–6308. 1631122 10.1073/pnas.89.14.6304 PMC49489

[71] Garza AG, Harris-Haller LW, Stoebner RA, Manson MD (1995) Motility protein interactions in the bacterial flagellar motor. Proc Natl Acad Sci USA 92:1970–1974. 7892209 10.1073/pnas.92.6.1970 PMC42404

[72] Marykwas DL, Schmidt SA, Berg HC (1996) Interacting components of the flagellar motor of *Escherichia coli* revealed by the two-hybrid system in yeast. J Mol Biol 256:564–576. 8604139 10.1006/jmbi.1996.0109

[73] Thomas D, Morgan DG, DeRosier DJ (2001) Structures of bacterial flagellar motors from two FliF-FliG gene fusion mutants. J Bacteriol 183:6404–6412. 11591685 10.1128/JB.183.21.6404-6412.2001 PMC100136

[74] Levenson R, Zhou H, Dahlquist FW (2012) Structural insights in the interaction between the bacterial flagellar motor proteins FliF and FliG. Biochem 51:5052–5060. 22670715 10.1021/bi3004582 PMC3384689

[75] Tang H, Braun TF, Blair DF (1996) Motility protein complexes in the bacterial flagellar motor. J Mol Biol 261:209–221. 8757288 10.1006/jmbi.1996.0453

[76] Kubori T, Shimamoto N, Yamaguchi S, Namba K, Aizawa S-I (1992) Morphological pathway of flagellar assembly in *Salmonella typhimurium* . J Mol Biol 226:433–446. 1640458 10.1016/0022-2836(92)90958-m

[77] Kubori T, Yamaguchi S, Aizawa S-I (1997) Assembly of the switch complex onto the MS ring complex of *Salmonella typhimurium* does not require any other flagellar proteins. J Bacteriol 179:813–817. 9006037 10.1128/jb.179.3.813-817.1997 PMC178764

[78] Suzuki T, Iino T, Horiguchi T, Yamaguchi S (1978) Incomplete flagellar structures in nonflagellate mutants of *Salmonella typhimurium* . J. Bacteriol. 33:904–915. 10.1128/jb.133.2.904-915.1978 PMC222103 342514

[79] Ueno T, Oosawa K, Aizawa S-I (1994) Domain structures of the MS ring component protein (FliF) of the flagellar basal body of *Salmonella typhimurium* . J Mol Biol 236:546–555. 8107139 10.1006/jmbi.1994.1164

[80] Katayama E, Shiraishi T, Ooswaw K, Baba N, Aizawa S-I (1996) Geometry of the flagellar motor in the cytoplasmic membrane of *Salmonella typhimurium* as determined by stereo-photogrammetry of quick-freeze deep-etch replica images. J. Mol. Biol. 255:458–475. 8568890 10.1006/jmbi.1996.0038

[81] Suzuki H, Yonejura K, Murata K, Hirai T, Oosawa K, Namba K (1998) A structural feature in the central channel of the bacterial flagellar FliF ring complex is implicated in type III protein export. J Struct Biol 124:104–114. 10049798 10.1006/jsbi.1998.4048

[82] Minamino T, Yamaguchi S, Macnab RM (2000) Interaction between FliE and FlgB, a proximal rod component of the flagellar basal body of *Salmonella* . J Bacteriol 182:3029–3036. 10809679 10.1128/jb.182.11.3029-3036.2000 PMC94486

[83] Zhao X, Zhang K, Boquoi T, Hu B, Motaleb MA, Miller KA, James ME, Charon NW, Manson MD, Norris SJ, Li C, Liu J (2013) Cryoelectron tomography reveals the sequential assembly of bacterial flagella in *Borrelia burgdorferi* . Proc Natl Acad Sci USA 110:14390–14395. 10.1073/pnas.1308306110 23940315 PMC3761569

[84] Hendrixson DR, DiRita VJ (2003) Transcription of σ^54^-dependent but not σ^28^-dependent flagellar genes in *Campylobacter jejuni* is associated with formation of the flagellar secretory apparatus. Mol Microbiol 50:687–702. 14617189 10.1046/j.1365-2958.2003.03731.x

[85] Joslin SN, Hendrixson DR (2009) Activation of the *Campylobacter jejuni* FlgSR two-component system is linked to the flagellar export apparatus. J. Bacteriol. 191:2656–2667. 10.1128/JB.01689-08 19201799 PMC2668382

[86] Boll JM, Hendrixson DR (2013) A regulatory checkpoint during flagellar biogenesis in *Campylobacter jejuni* initiates signal transduction to activate transcription of flagellar genes. mBio:e00432–13. 10.1128/mBio.00432-13 24003178 PMC3760246

[87] Ramakrishnan G, Zhao J-L, Newton AN. 1994 Multiple structural proteins are required for both transcriptional activation and negative autoregulation of *Caulobacter crescentus* flagellar genes. J. Bacteriol. 176:7587–7600. 8002583 10.1128/jb.176.24.7587-7600.1994 PMC197216

[88] Mohr CD, MacKichan JK, Shapiro L. 1998 A membrane-associated protein, FliX, is required for an early step in *Caulobacter* flagellar assembly. J. Bacteriol. 180:2175–2185. 9555902 10.1128/jb.180.8.2175-2185.1998 PMC107146

[89] Boyd CH, Gober JW. 2001 Temporal regulation of genes encoding the flagellar proximal rod in *Caulobacter crescentus* . J. Bacteriol. 183:725–735. 11133968 10.1128/JB.183.2.725-735.2001 PMC94930

[90] Muir RE, O’Brien TM, Gober JW. 2001 The *Caulobacter crescentus* flagellar gene, *fliX*, encodes a novel trans-acting factor that couples flagellar assembly to transcription. Mol. Microbiol. 39:1623–1637. 11260478 10.1046/j.1365-2958.2001.02351.x

[91] Dasgupta N, Wolfgang MC, Goodman AL, Arora SK, Jyot J, Lory S, Ramphal R. 2003 A four-tiered transcriptional regulatory circuit controls flagellar biogenesis in *Pseudomonas aeruginosa* . Mol. Microbiol. 50:809–824. 14617143 10.1046/j.1365-2958.2003.03740.x

[92] Kenjale R, Wilson J, Zenk SF, Saurya S, Picking WL, Picking WD, Blocker A (2005) The needle component of the type III secretion of *Shigella* regulates the activity of the secretion apparatus. J Biol Chem 280:42929–42937. 16227202 10.1074/jbc.M508377200

[93] Deane JE, Roversi P, Cordes FS, Johnson S, Kenjale R, Daniell S, Booy F, Picking WD, Picking WL, Blocker AJ, Lea SM (2006) Molecular model of a type III secretion system needle: implications for host-cell sensing. Proc Natl Acad Sci USA 103:12529–12533. 16888041 10.1073/pnas.0602689103 PMC1567912

[94] Diepold A, Wiesand U, Amstutz M, Cornelis GR (2012) Assembly of the *Yersinia* injectisome: the missing pieces. Mol Microbiol 85:878–892. 10.1111/j.1365-2958.2012.08146.x 22788867

[95] Yasbin RE, Young FE (1974) Transduction in *Bacillus subtilis* by bacteriophage SPP1. J Virol 14:1343–1348. 4214946 10.1128/jvi.14.6.1343-1348.1974 PMC355660

[96] Konkol MA, Blair KM, Kearns DB (2013) Plasmid-encoded ComI inhibits competence in the ancestral 3610 strain of *Bacillus subtilis* . J Bacteriol 195:4085–4093. 10.1128/JB.00696-13 23836866 PMC3754741

[97] Guérout-Fleury A-M, Frandsen N, Stragier P (1996) Plasmids for ectopic integration in *Bacillus subtilis* . Gene 180:57–61. 8973347 10.1016/s0378-1119(96)00404-0

[98] Patrick JE, Kearns DB (2008) MinJ (YvjD) is a topological determinant of cell division in *Bacillus subtilis* . Mol Microbiol 70:1166–1179. 10.1111/j.1365-2958.2008.06469.x 18976281

[99] Gibson DG, Young L, Chuang R-Y, Venter JC, Hutchinson CA III, Smith HO (2009) Enzymatic assembly of DNA molecules up to several hundred kilobases. Nat Meth 6:343–345. 10.1038/nmeth.1318 19363495

[100] Ben-Yehuda S, Rudner DZ, Losick R (2003) RacA, a bacterial protein that anchors chromosomes to the cell poles. Science 299:532–536. 12493822 10.1126/science.1079914

[101] Kearns DB, Losick R (2003) Swarming motility in undomesticated *Bacillus subtilis* . Mol. Microbiol. 49:581–590. 12864845 10.1046/j.1365-2958.2003.03584.x

[102] Chan JM, Guttenplan SB, Kearns DB (2014) Defects in the flagellar motor increase synthesis of poly-γ-glutamate in *Bacillus subtilis* . J. Bacteriol. 196:740–753. 10.1128/JB.01217-13 24296669 PMC3911173

