# Supplementary material for: Functional Activation of the Flagellar Type III Secretion Export Apparatus
Source: PLoS Genet. 2015 Aug 5;11(8):e1005443. doi: 10.1371/journal.pgen.1005443 (PMC4526659; doi:10.1371/journal.pgen.1005443)
Supplement: S1 Table — (DOCX) [file pgen.1005443.s007.docx]

**Table S1: Plasmids**

| Plasmid | Genotype |
| --- | --- |
| pAH25 | *amyE::spec (erm) amp* (Blair et al., 2008) |
| pAP44 | *P_flache_^sob21^ mls amp* |
| pAP46 | *P_flache_^sob6^ mls amp* |
| pAP50 | *amyE::P_flache_^sob6^-lacZ spec* |
| pAP60 | *amyE::P_flache_-fliO spec amp* |
| pAP61 | *amyE::P_flache_-fliO^sob22^ spec amp* |
| pAP69 | *amyE::P_flache_-fliP spec amp* |
| pAP70 | *amyE::P_hyspank_-fliP spec* *amp* |
| pAP71 | *amyE::P_flache_-fliP^sob22^ spec amp* |
| pAP80 | *fliG^sob28^ mls amp* |
| pAP85 | *amyE::P_hyspank_-fliO spec* *amp* |
| pAP94 | *ΔcspB mls amp* |
| pDG1728 | *amyE::lacZ spec amp* (Guérout-Fleury et al., 1996) |
| pDP332 | *ΔfliO mls amp* |
| pDP346 | *ΔfliP mls amp* |
| pDR111 | *amyE::P_hyspank_ spec amp* (Ben-Yehuda et al., 2003) |
| PMiniMAD | *ori^BsTs^ mls amp* (Patrick and Kearns, 2008) |
| pSG32 | *ΔfliM mls amp* (Guttenplan and Kearns, 2013) |
