# Supplementary material for: Functional Activation of the Flagellar Type III Secretion Export Apparatus
Source: PLoS Genet. 2015 Aug 5;11(8):e1005443. doi: 10.1371/journal.pgen.1005443 (PMC4526659; doi:10.1371/journal.pgen.1005443)
Supplement: S2 Table — (DOCX) [file pgen.1005443.s008.docx]

**Table S2: Primers**

| Primer | Sequence |
| --- | --- |
| 321 | AGGAGGAATTCTTGCTGACCGTGTCGGCATTACCC |
| 322 | CTCCTGGATCCTCAGTTTTTTTCACCCTCAATATCCT |
| 788 | ATAATCCTGAGTAAATGCAGAAGATAA |
| 1229 | AGGAGGAATTCTAGAGCCGCCTGATGCCAAAAAC |
| 1448 | AAGAAGATTGGCTGCCCAGAA |
| 1449 | TCCGAAGATACCGCTGTTCAAG |
| 1450 | GGCAGAGAACCAACACCTGAA |
| 1451 | TCCAGAGATACCGGCTCTTGA |
| 1482 | ctcctggatccctcactcgttttgtttttcatttgc |
| 1560 | TGTTGCGAGCGGAGATACA |
| 1561 | TTTGCGCCAATTCTGTCATC |
| 1562 | GTTGGGATTGCTGACGTGAA |
| 1563 | TCCGGAAGCATGACATGAAC |
| 1564 | GCAGGTCTTGCGATCTCTGAA |
| 1565 | CACGTTGAAGGATCGCATGAG |
| 1569 | AGGAGGAATTCCGGGATTGTTACTTTTGCCAACAG |
| 1572 | CTCCTGTCGACCTCGATAATACTTCCGGCAGAAAG |
| 1596 | AAACACTGCGTCGCTATCAAC |
| 1597 | GGCTGAACGGAAACAACAATC |
| 1598 | agcaaatcggtttaggttcat |
| 1599 | GTCCCGTCACCTGTATCAAT |
| 1601 | CACACTTTTCCCGACCTTGAT |
| 1602 | AGCATGCCCACGAATTGAT |
| 1603 | AATGGATTCGGCGTAACCTC |
| 1604 | ACATCAACAGGCGGTCCA |
| 1605 | CAACCACTGGTGCGTTCAA |
| 1648 | AGGAGGAATTCACAGTATGTCGAAGAAAAGCTCGG |
| 1652 | GAACGAACAGGTGCAAACGA |
| 1653 | TCGGCTCATTCATGTCATCC |
| 1654 | ATCGGGGCATAGGAGGAGTT |
| 1655 | CCGTTTTTGTTTCGGCATTT |
| 1656 | TGCGTTTCCTGAACCGTATG |
| 1657 | GCGTCACTTTGTTCCACGAC |
| 1658 | TGTCAATGCCCACCATTTGT |
| 1659 | CAAACTGTTCATCGCCGAAA |
| 1662 | ACGCCTGAATTTGCCAGAGT |
| 1663 | TCGCTTCATCCACAATCCAG |
| 1692 | AGGAGGAATTCCTGATGATGAAATGCTGGTGAAAG |
| 1693 | CTCCTCTCGAGCTTTTTCAACAGTCGTACACCCT |
| 1694 | AGGAGCTCGAGTCTGAAGGAAAGAAGAAAGGCCCA |
| 1695 | CTCCTGGATCCAATTGAATTCATCTCTACATTACCCT |
| 1921 | AGGAGGAATTCAGGTACTTATATCAAGGTACTAAACAA |
| 1922 | CTCCTGCATGCTCACCCTCAATATCCTTGTCGAGA |
| 2290 | aggaggaattccgactgttgaaaaagagtcaat |
| 2291 | ctcctctcgagcgaacttacgttttccggatc |
| 2292 | aggagctcgagatgctgcctccagttatgatt |
| 2293 | aggagctcgagatgctgcctccagttatgatt |
| 2460 | aggaggaattcaggtacttatatcaaggtactaa |
| 2461 | ctcctctcgagtcaccctcaatatccttgtcgag |
| 3177 | ctaattcaaggcgtgtctcac |
| 3180 | gcggtattccgtatgtcaag |
| 3217 | AGGAGCTCGAGtgaccgtgtgcttgaagcaatcaa |
| 3218 | CTCCTGGATCCtttccggatcgcttgaactgaaaat |
| 3449 | AGGAGCTCGAGagcaacttgaagagttaaaacaaaa |
| 3450 | CTCCTGGATCCaaagctctgaagcaaagatttcacaa |
| 3451 | AGGAGGCTAGCagcaacttgaagagttaaaacaaaa |
| 3473 | CTCCTGCATGCattctgaactcacgtttagcaccta |
| 3476 | CTCCTGAATTCtgcccatgggcgttatgtatgtc |
| 3628 | tcgcaatgtggttattcttcgatga |
| 3629 | gatggcaacgactgcgataacag |
| 3632 | CTCCTGTCGACtaacctgttgtacaagcaggagga |
| 3733 | catggatccgatcagaccag |
| 3741 | AGGAGGCTAGCgaccgtgtgcttgaagcaatcaa |
| 3742 | CTCCTGCATGCtttccggatcgcttgaactgaaaat |
| 3758 | gtggcgagcgtcttgatgtc |
| 3759 | accagccgtctaccaaaacaa |
| 3944 | Ctcctgtcgacgaagcgggatattgcaaactaa |
| 3945 | ctcctggatccgttgaaccattttactttaccttc |
| 3946 | aggagggatcccaagctgctaacgttactaaag |
| 3947 | ctcctctcgagcaacgtatctttctgaatccg |
| 4086 | ctgcagccctggcgaatg |
| 4087 | gccattcgccagggctgcagttcttccaatctgaaagacgtt |
| 4088 | ctggtctgatcggatccatgtgctaatgtcgacttgactca |
| 4089 | ctggtctgatcggatccatgaacgcttaaattcactattcttg |
| 4090 | gccattcgccagggctgcagtgtgaccatactcctatctatg |
| 4560 | aggaggctagcgtgaaatctttgcttcagagc |
| 4561 | ctcctgcatgcgcaggaaataagtcaataattgaa |
