# Supplementary material for: Functional Activation of the Flagellar Type III Secretion Export Apparatus
Source: PLoS Genet. 2015 Aug 5;11(8):e1005443. doi: 10.1371/journal.pgen.1005443 (PMC4526659; doi:10.1371/journal.pgen.1005443)
Supplement: S3 Table — (DOCX) [file pgen.1005443.s009.docx]

**Table S3: β-galactosidase activities to support figure 2B^a^**

| Genotype | *amyE::P_hag_-lacZ* (MU) |
| --- | --- |
| wild type | 116.99 ± 35.66 (DS9461) |
| *flgM* | 263.32 ± 20.59 (DK313) |
| *swrA* | 56.57 ± 23.77 (DK288) |
| *swrA flgM* | 280.82 ± 41.07 (DK318) |
| *swrB* | 40.04 ± 4.15 (DK289) |
| *swrB flgM* | 159.17 ± 18.66 (DK319) |

^a^All cultures were grown to exponential growth phase in LB medium. All values are the average of three replicas (Miller units ± standard deviation).
