# Supplementary material for: Functional Activation of the Flagellar Type III Secretion Export Apparatus
Source: PLoS Genet. 2015 Aug 5;11(8):e1005443. doi: 10.1371/journal.pgen.1005443 (PMC4526659; doi:10.1371/journal.pgen.1005443)
Supplement: S4 Table — (DOCX) [file pgen.1005443.s010.docx]

**Table S4: β-galactosidase activities to support figure 6^a^**

| Genotype | *amyE::P_flache_-lacZ* (MU) | *amyE::P_flache_^sob21^-lacZ (MU)* | *amyE::P_flache_^sob6^-lacZ (MU)* |
| --- | --- | --- | --- |
| wild type | 350.69 ± 27.63 (DS611) | 517.49 ± 98.17 (DS1426) | 274.05 ± 62.83(DS9120) |
| *swrA* | 110.60 ± 5.76 (DK284) | 355.16 ± 57.84 (DK1129) | 143.63 ± 8.01 (DK1116) |
| *swrB* | 289.55 ± 47.36 (DK285) | 825.55 ± 20.05 (DK1130) | 5.66.05 ± 50.94 (DK1117) |

^a^All cultures were grown to exponential growth phase in LB medium. All values are the average of three replicas (Miller units ± standard deviation).
