# Supplementary material for: Functional Activation of the Flagellar Type III Secretion Export Apparatus
Source: PLoS Genet. 2015 Aug 5;11(8):e1005443. doi: 10.1371/journal.pgen.1005443 (PMC4526659; doi:10.1371/journal.pgen.1005443)
Supplement: S5 Table — (DOCX) [file pgen.1005443.s011.docx]

**Table S5: Supplemental strains**

| Strain | Genotype |
| --- | --- |
| DK21 | *swrB::Tn10 spec amyE::P_flache_^sob6^-lacZ cat* |
| DK838 | *ΔfliM ΔfliG sob28 amyE::P_hag_-hag^T209C^ spec* |
| DK839 | *ΔfliM amyE::P_hag_-hag^T209C^ spec* |
| DK1966 | *swrB::tet amyE::P_flache_-lacZ cat* |
| DK1978 | *swrB::tet sob28 amyE::P_hag_-hag^T209C^ spec* |
| DS793 | *amyE::Pflache-lacZ* |
| DS1107 | *swrB::Tn10 spec* |
| DS1461 | *swrB::tet amyE::Pflache-lacZ cat* |
| DS7063 | *swrB::Tn10 spec sob6* |
| DS9974 | *swrB::Tn10 spec sob6 amyE::Pflache-lacZ cat* |
